# Population pharmacokinetics/pharmacodynamics and safety of YPEG-rhGH in elderly subjects

**DOI:** 10.3389/fphar.2025.1651323

**Published:** 2025-11-25

**Authors:** Yajie He, Jingwen Hu, Xin Zeng, Qianfeng Yang, Qiuyun You, Jiangeng Huang, Yu Zhang, Luqin Si, Xuejia Zhai

**Affiliations:** 1 Department of Pharmacy, Union Hospital, Tongji Medical College, Huazhong University of Science and Technology, Wuhan, Hubei, China; 2 Hubei Key Laboratory of Wudang Local Chinese Medicine Research, Hubei University of Medicine, Wuhan, Hubei, China; 3 School of Pharmacy, Tongji Medical College, Huazhong University of Science and Technology, Wuhan, Hubei, China; 4 School of Pharmacy, Hubei University of Chinese Medicine, Wuhan, Hubei, China; 5 Hubei Shizhen Laboratory, Wuhan, Hubei, China; 6 Hubei Health Industry Development Research Center, Hubei University of Chinese Medicine, Wuhan, Hubei, China

**Keywords:** Y-shape branched PEGylation recombinant human growth hormone, population pharmacokinetic/pharmacodynamic analysis, long-acting growth hormone, growth hormone deficiency, the indirect response model

## Abstract

Y-shape branched PEGylated recombinant human growth hormone (YPEG-rhGH) is a suitable drug for the treatment of growth hormone deficiency. The aim of this study was to establish a population pharmacokinetics/pharmacodynamics (PopPK/PD) model of YPEG-rhGH in the elderly. The safety and tolerability of the drug were investigated, and the possibility of flexible dosing regimen was explored. A total of 16 healthy elderly subjects and 36 healthy adults participated in the PopPK analysis. Only elderly subjects were included in the PopPK/PD analysis. After subcutaneous injection of the drug, serum samples were collected to analyze the time course of YPEG-rhGH concentrations and insulin-like growth factor-1 (IGF-1) levels. Additionally, the safety and tolerability of the investigational drug were evaluated. Results show that the pharmacokinetics of serum YPEG-rhGH after subcutaneous injection can be better described by a two-compartment model with first-order absorption and nonlinear elimination. The indirect response model (IDR) demonstrated good predictive capability for the relative baseline ratio of IGF-1 levels in elderly subjects following YPEG-rhGH administration. The magnitude of the increase in the relative baseline ratio of IGF-1 decreased with increasing weight, and increasing age marginally reduced the increase in the baseline ratio of YPEG-rhGH-increased IGF-1 in elderly people, but the effect decreased with increasing dosing intervals. All reported adverse reactions were mild. In the final model, AGE was identified as a significant covariate for K_a_, V_1_/F, and V_max_, while WEIGHT significantly influenced V_max_. The IDR model was consistent with the PK/PD profile of elderly subjects. The safety and tolerability of YPEG-rhGH injections in elderly subjects were favorable.

**Clinical Trial Registration:** identifier CTR20230176.

## 1 Introduction

Growth hormone (GH) is a polypeptide consisting of 191 amino acids secreted by the anterior pituitary in humans. It possesses anabolic and growth-promoting properties (Melmed, 2019). Adult growth hormone deficiency (GHD) is a rare condition. In patients with GHD, the synthesis and secretion of GH by the anterior pituitary are impaired, leading to either partial or complete deficiency of GH (Hoffman et al., 2025). This may affect the metabolism of proteins, lipids and carbohydrates, leading to metabolic disorders, dyslipidemia, elevated total cholesterol, concentric obesity, skeletal muscle and lean body mass loss, prone to insulin resistance; left ventricular dysfunction, abnormal cardiac index, *etc.*, increased risk of cardiovascular and cerebrovascular diseases; reduced bone mineral density and increased bone fragility lead to osteoporosis and increase the risk of fracture. Additionally, patients may experience reduced physical strength and energy, diminished muscle strength and exercise capacity, and low mood, all of which contribute to a decreased quality of life (Ratku et al., 2022; Wydra et al., 2023; Yuen et al., 2023; Fukuoka et al., 2025).

Currently, there is a lack of epidemiological data on GHD in the elderly population, but with advances in medical technology and a decreasing birth rate, the proportion of elderly individuals is increasing. The growth rate of the global population over 65 years old has exceeded that of any other age group. The United Nations predicts that by 2050, one-sixth of the world’s population will be over 65 years old, with China’s elderly population reaching 26% (Cai et al., 2022). This demographic shift suggests that the proportion of elderly individuals with GHD will continue to rise. With the increase of age, the organs of the human body are gradually aging, and their functions decline due to the altered structures, resulting in a decrease in learning and memory ability, a change in metabolic spectrum, and an increase in the risk of cardiovascular disease, osteoporosis, sarcopenia and other diseases. Among elderly populations, the clinical manifestations of GHD tend to be more pronounced compared to younger adults. This population frequently presents with multiple comorbidities, and polypharmacy may lead to drug interactions that potentially induce or exacerbate adverse drug reactions. These factors collectively exacerbate age-related health challenges and contribute significantly to the growing burden on global healthcare systems. Consequently, addressing GHD in the elderly population is emerging as a critical public health challenge.

Previous clinical studies and practices have demonstrated that rhGH therapy can increase bone mass, improve blood lipids, metabolic status and body composition in patients with GHD, and enhance the quality of life of patients (Appelman-Dijkstra et al., 2016; Yuen et al., 2019; Yang et al., 2023). IGF-1 is an important biomarker reflecting growth hormone activity. It has been established in the literature that IGF-1 correlates with clinical treatment endpoints for GHD, such as bone mineral density, bone mass, muscle mass, and muscle strength (Ascenzi et al., 2019; Dixit et al., 2021; Fang et al., 2023). Growth hormone replacement therapy typically requires long-term or lifelong administration, with most of the listed rhGH products requiring daily injections. This daily administration regimen can reduce patient adherence and consequently diminish the therapeutic benefits of the treatment (Mancini et al., 2019; Oi-Yo et al., 2024). The investigational drug was YPEG-rhGH. The Y-shape branched polyethylene glycol with a molecular weight of 40 KDa was covalently linked to the growth hormone protein, followed by purification and formulation. YPEG-rhGH was developed as a therapeutic option for endogenous growth hormone deficiency and demonstrates comparable efficacy with extended dosing intervals, enhancing patient adherence through reduced administration frequency compared to conventional growth hormone therapies, thereby optimizing therapeutic outcomes.

The primary objectives of this study were to comprehensively assess the pharmacokinetics, pharmacodynamics, and safety of YPEG-rhGH in a geriatric population (age ≥65 years). This investigation contributes significantly to elucidating the dose-exposure relationship in special populations, while establishing a solid foundation for further clinical development and regulatory evaluation.

## 2 Materials and methods

### 2.1 Ethics

This clinical trial protocol and all relevant amendments were reviewed and approved by the ethics committee of the Union Hospital of Tongji Medical College, Huazhong University of Science and Technology. The clinical trial protocol was formulated in accordance with the ethical and scientific principles stipulated in the Helsinki Declaration of the 1964 World Medical Association Joint Conference and its amendments and the Good Clinical Practice standards of China National Medical Products Administration. Before screening, the researchers gave informed consent to the subjects and obtained the signed and dated informed consent of the subjects. The registration number of the clinical trial is CTR20230176.

### 2.2 Subjects

Inclusion criteria: (1) individuals were aged 65–80 years (including 65 years old, excluding 80 years old); (2) both male and female can participate, and female subjects must be postmenopausal for more than 1 year; (3) body mass index (BMI) of 18.0–30.0 kg/m^2^; (4) IGF-I level <0 SD; (5) if other endocrine hormones are insufficient, replacement therapy should be received before screening, and sufficient and stable treatment for more than 3 months as judged by the researcher should be achieved before the baseline; (6) subjects were able to cooperate with the completion of scheduled visits, research programs and laboratory examinations and other test procedures; (7) the subjects could understand and sign the informed consent.

Exclusion criteria: (1) individuals with severe allergic constitution or allergies to test drugs and excipients; (2) growth hormone or growth hormone secretagogue drugs were used within 6 months before screening; (3) individuals with significant liver and kidney dysfunction; (4) thyroid function: thyroid stimulating hormone (TSH) > 10 ng/mL; (5) individuals with mild to moderate hypertension were still≥140/90 mmHg after drug control, or individuals with severe hypertension; (6) individuals with severe heart disease, including the New York heart association cardiac function class (NYHA) > 2, serious cardiac arrhythmia, unstable angina or myocardial infarction in the past 6 months; (7) intracranial hypertension, history of seizures, stroke, subarachnoid hemorrhage and other medical history; (8) acute critical illness; (9) participated in any interventional study and received the study intervention (as a subject) within 3 months prior to screening; (10) those who have other factors deemed unsuitable for participation in this study by the researchers.

### 2.3 Study design and treatments

This was a single-center, open, single-arm, non-randomized study of safety and human pharmacokinetics (PK)/pharmacodynamics (PD) in the elderly population, and did not involve the allocation of patient treatment groups. A total of 16 healthy elderly subjects participated in the project.

Screening visits were conducted 4 weeks before the start. Eligible subjects were initiated at baseline with YPEG-rhGH (lot no. 202204M02; Xiamen Amoytop Biotech Co., Ltd., Xiamen, China). The dose was 30 μg/kg, once every 2 weeks, a total of 23 weeks of administration (12 injections), withdrawal observation for 5 weeks (to 28 weeks). In principle, no dose adjustment or discontinuation was performed, except for safety reasons. Each dose was given in the morning, and the dose was repeated at different points within the same month.

Data from 36 healthy adult men from another clinical trial were included in the analysis, which has been reported previously (Liang et al., 2022). A table comparing the key differences and similarities in the PopPK/PD analyses between the elderly and adult trials across the two studies is presented in Table 1. Healthy adult subjects were administered the drug in two phases. In the first phase, subjects were randomly assigned to receive daily subcutaneous injections of the positive control drug (Saizen^®^, Serono, Switzerland) at doses of either 0.1 IU/kg or 0.15 IU/kg for seven consecutive days. Following a 14-day washout period, the study proceeded to the second phase, where subjects were divided into YPEG-rhGH dose groups and administered a single subcutaneous injection of the investigational drug at one of five dose levels (10 μg/kg, 30 μg/kg, 60 μg/kg, 120 μg/kg, and 200 μg/kg).

**TABLE 1:** Key differences and similarities between the elderly trial and the adult trial included in the PopPK/PD analysis.

Category	Elderly study	Adult study (Liang et al., 2022)
Trial design	Single-arm, open-label, single-center, multiple-dose study	Phase-1, multicenter, randomized, single-dose (10–200 μg/kg) with short daily rhGH comparator
Population	Healthy elderly subjects (≥65 years, n = 16; 75% female)	Healthy young adult male volunteers (n = 36, 100% male)
Dosing regimen	YPEG-rhGH 30 μg/kg SC, once every 2 weeks, for 23 weeks (12 injections)	Single SC doses of YPEG-rhGH (10, 30, 60, 120, 200 μg/kg); short-term daily rhGH control
PK/PD sampling schedule	Pre-dose, 11 ± 3 h, 96 ± 3 h post-dose, pre-next dose at 1st and 12th injection, and 11 ± 3 h after 4th injection	Multiple post-dose time points (frequent sampling in first 96 h for PK/PD assessment)
Geographic location	Single center	Multiple centers across China (12 trial sites)
Laboratory assays	IGF-1 measured by Siemens ELISA kit (Catalog L2KGF2), PK assay validated by sponsor laboratory	IGF-1 measured by Siemens ELISA kit (Catalog L2KGF2), PK assay validated by the same sponsor laboratory
Inclusion criteria	Age ≥65, BMI 18–30 kg/m^2^, IGF-1 < 0 SD, stable replacement therapy if applicable	Age 18–45, BMI within normal range, healthy status confirmed by medical exam
Exclusion criteria	Uncontrolled hypertension (≥140/90 mmHg), significant cardiac disease, renal/hepatic impairment, recent MI/stroke, abnormal ECG/echocardiography	Any chronic illness, abnormal labs, prior GH treatment, other standard healthy volunteer exclusions

Blood samples were collected to determine the PK/PD profile of YPEG-rhGH. IGF-1 was determined by enzyme linked immunosorbent assay (ELISA) kit.

### 2.4 Pharmacometric modeling strategy

All data were processed using SAS software (Version 9.4, SAS Institute Inc., United States). The nonlinear mixed-effects model in Phoenix NLME (Version 8.1, Certara United States, Inc.) was employed to develop the PopPK/PD model. Exploratory data analysis was conducted, and model selection was performed by comparing objective function values and goodness-of-fit plots among various models. After identifying the base model, a stepwise approach involving forward inclusion and backward elimination was used to evaluate covariates with significant effects on YPEG-rhGH PopPK. Once the final PK model was established, the PopPK/PD model was further developed. The parameters of the PopPK/PD model were estimated using the first order conditional estimation with η-ε interaction option (FOCEI). The PopPK/PD model was validated using goodness-of-fit (GOF) diagnostic plots and prediction- and variability-corrected visual predictive checks (pvcVPC). Data organization and graphical representation of model results were performed using R software (Version 4.4.1, Lucent Technologies, United States).

#### 2.4.1 Base model

**FIGURE 1:**
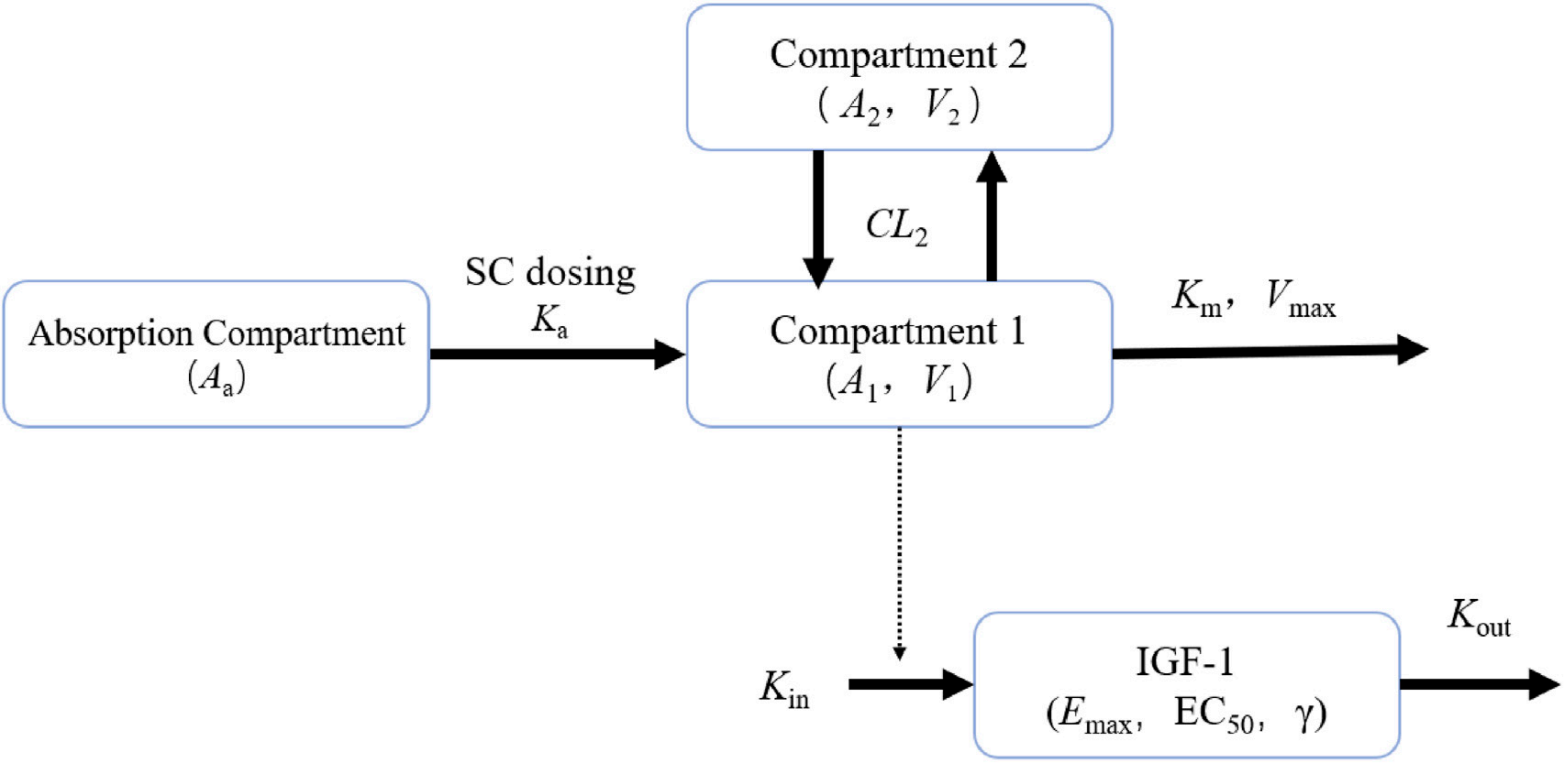
PK/PD model plots of healthy adult subjects and elderly subjects given YPEG-rhGH A_a_ represents the drug amount in the absorption compartment, K_a_ denotes the first-order absorption rate constant describing drug transfer from the absorption compartment into the systemic circulation. A_1_ and A_2_ correspond to the drug amounts of YPEG-rhGH in the central and peripheral compartments, respectively. V_1_ indicates the apparent volume of distribution of YPEG-rhGH in the central compartment. V_max_ represents the maximum clearance rate, while K_m_ signifies the concentration to reach half of maximum clearance. CL_2_ denotes the intercompartmental clearance, and V_2_ refers to the apparent volume of distribution of YPEG-rhGH in the peripheral compartment. K_in_ represents the zero-order production rate of IGF-1, and K_out_ is the first-order elimination rate constant of IGF-1. E_max_ characterizes the maximum stimulatory effect of YPEG-rhGH on IGF-1 production rate, and EC_50_ indicates the YPEG-rhGH concentration required to reach half of the maximum effect. γ represents the Hill coefficient, a shape factor of the sigmoid function that determines the steepness of the sigmoid curve.

Based on the completed PK/PD model analysis of YPEG-rhGH in healthy subjects, we utilized a two-compartment model with nonlinear elimination as the structural model to fit the observed PK data of YPEG-rhGH. The IDR model of YPEG-rhGH-induced IGF-1 generation was employed to characterize the IGF-1 kinetic profile following YPEG-rhGH administration. The model framework used for the PK/PD analysis of YPEG-rhGH and IGF-1 data in this study is illustrated in Figure 1.

#### 2.4.2 Covariate analysis

The stepwise covariate method (SCM) was used to establish the covariate model. SCM consists of two processes: forward addition and backward elimination. During the forward addition step, each covariate potentially related to PK parameters was sequentially incorporated into the base model’s PK parameters. The selection of covariates was based on a combination of objective function value (OFV) changes and graphical evaluation. Covariates demonstrating statistically significant influence (ΔOFV >6.64, p < 0.01, df = 1) and producing the largest reduction in OFV were preferentially incorporated into the model, which then served as the base model for subsequent forward addition steps. A full-size model was obtained until there were no other significant covariates.

For the backward elimination step, each previously included covariate was systematically removed from the model. A covariate was retained in the final model only if its removal resulted in an OFV increase of at least 10.83 (p < 0.001, df = 1). The elimination process continued until all remaining covariates met the stringent criterion of p < 0.001, at which point the model was considered final.

The continuous covariate was added to the model, as shown in Equation 1:

θi =θTV×Cov_i / Cov_median θx (1)

θ_i_ is the PK parameter value of the *i*th individual, and θ_TV_ is the population typical value. Cov__i_ is the covariate value of the *i*th individual, Cov__median_ is the median value of this covariate, and θ_x_ is the coefficient of influence of the covariate on the parameter.

The categorical covariate (such as sex) was incorporated into the model using Equation 2:

θi =θTV×e θx (2)

The θ_TV_ is the typical value of the population, and θ_x_ is the influence coefficient of the covariates on the parameters. The values of θ_x_ are different according to the classification of covariates.

#### 2.4.3 Model evaluation

During the modeling process, it is crucial to evaluate model stability, check for ill-conditioning, examine covariance outputs, and identify extreme parameter correlations (e.g., correlation coefficient >0.95) or condition numbers exceeding 1,000. The GOF of different models was evaluated by the following methods: the variation of OFV, the visual display of various diagnostic methods, parameter prediction precision and rationality.

The pvcVPC method was employed for model predictive performance diagnostics. The success rate of Bootstrap 1,000 times was used to verify the stability of the model, and the Bootstrap parameters were used to verify the prediction precision of the model parameters.

#### 2.4.4 Simulations based on the PopPK/PD model

A PK/PD model was developed to simultaneously fit the serum concentration of YPEG-rhGH and the relative baseline ratio level of IGF-1 after the administration of YPEG-rhGH, and based on the model, the inter-individual variation (IIV) of the parameters, and residual error, the PK/PD changes in different scenarios were simulated according to the available formulation specifications. Different dosing scenarios were fitted with one specification unit dose of drug given at a time to compare the PK and PD differences between different specifications, different dosing cycles and different titration schemes.

### 2.5 Safety assessments

The safety and tolerability of the YPEG-rhGH were evaluated. All eligible subjects who received ≥1 dose of test drug were included in the safety population. Safety assessment is based on adverse events (AEs) or serious adverse events, relevant vital signs and physical examination, and clinical laboratory results including complete blood count (CBC), urinalysis, and blood chemistry panel. The severity of adverse events and laboratory examinations was evaluated by NCI-CTCAE V5.0.

## 3 Results

### 3.1 Demographics

The study involved the screening of 171 subjects, of whom 155 were excluded based on failure to meet inclusion criteria or meeting exclusion criteria, and 16 subjects were ultimately enrolled. Among the 155 screening failures, more than 30% were due to unqualified vital signs. The primary reason for screening failure was blood pressure values meeting the exclusion criteria (≥140/90 mmHg). This finding correlates with the high prevalence of hypertension among residents aged over 60 years in China (the survey showed that 67.8% (Long et al., 2022)). Approximately 20% of the subjects failed in screening due to unqualified electrocardiogram or unqualified echocardiography. No early withdrawals occurred, and all 16 subjects (100.0%) completed the clinical study. 36 healthy male subjects from a separate clinical trial were also included in this analysis. Table 2 gives the characteristics of the subjects.

**TABLE 2:** Population characteristics.

Category	Group	Elderly subjects (TB2208GH) n = 16 Mean (SD) Median [Min, Max]	Healthy adult subjects (TB1010GH) n = 36 Mean (SD) Median [Min,Max]
Gender	Female	12	0
	Male	4	36
Age (year)		67.44 (3.01) 66.00 [65.00, 74.00]	30.78 (6.62) 29.00 [21.00, 44.00]
Weight (kg)		60.54 (7.31) 59.00 [51.10, 76.10]	65.08 (6.16) 66.40 [52.40, 79.60]
Body mass index, BMI (kg/m^2^)		23.70 (2.30) 23.62 [20.27, 27.64]	23.08 (2.12) 23.65 [19.50, 26.90]
Alanine aminotransferase, ALT (U/L)		16.06 (4.40) 15.00 [10.00, 25.00]	16.86 (8.60) 14.50 [7.00, 46.00]
Aspartate aminotransferase, AST (U/L)		22.38 (3.22) 22.50 [17.00, 28.00]	21.69 (9.31) 19.00 [12.00, 63.00]
Total protein,TP (g/L)		68.43 (4.45) 67.10 [61.10, 77.10]	71.81 (3.90) 71.50 [64.00, 80.00]
Albumin, ALB (g/L)		39.08 (1.60) 38.85 [36.10, 41.60]	45.72 (2.78) 45.50 [41.00, 51.00]
Total bilirubin, TBIL (μmol/L)		9.86 (4.85) 9.10 [4.80, 22.10]	12.76 (4.22) 11.95 [6.50, 23.40]
Alkaline phosphatase, ALP(U/L)		71.50 (18.38) 69.00 [45.00,110.00]	66.94 (15.86) 67.00 [37.00,100.00]
Creatinine clearance, CL_CR_ (mL/min)		76.66 (11.06) 75.78 [62.55, 99.45]	120.80 (18.47) 119.06 [90.56,160.52]
Total cholesterol, TC (mmol/L)		5.39 (1.17) 5.27 [3.76, 7.33]	3.89 (0.73) 3.74 [2.57, 5.76]
Triglyceride, TG (mmol/L)		1.62 (0.87) 1.35 [0.73, 4.19]	0.88 (0.41) 0.81 [0.26, 2.04]
High-density lipoprotein, HDL (mmol/L)		1.54 (0.34) 1.46 [0.97, 2.22]	1.26 (0.27) 1.23 [0.79, 2.12]
Low-density lipoprotein,LDL (mmol/L)		2.97 (0.82) 2.70 [1.85, 4.32]	2.26 (0.65) 2.22 [0.98, 3.76]

### 3.2 Pharmacokinetics

A total of 884 PK blood samples were provided by 52 subjects included in the analysis, of which 71 samples were below the quantification limit (BQL). BQL samples accounted for less than 20% of the total sample after the first dose, and were therefore excluded from the analysis. Consequently, a total of 813 PK samples were utilized to establish the PopPK model of YPEG-rhGH in elderly subjects and healthy adults.

**FIGURE 2:**
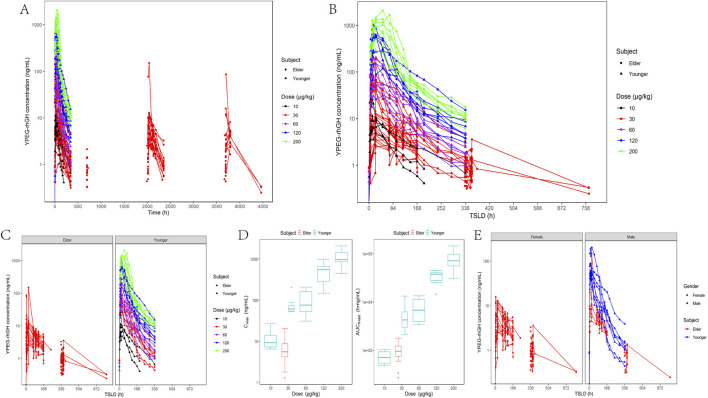
*In vivo* PK characterization of YPEG-rhGH in adult healthy and elderly subjects. **(A,B)** Serum concentration - time curve of YPEG-rhGH after administration (semi-log coordinate plot). TSLD:time since the last dose. **(C)** Serum concentration - time curve of YPEG-rhGH in different age groups (semi-log plot). **(D)** C_max_ and AUC_0-last_ box plots after first administration of different doses of YPEG-rhGH. **(E)** Serum concentration - time curve of YPEG-rhGH in different genders after 30 μg/kg dose of YPEG-rhGH (semi-log coordinate plot).

The PK profile of YPEG-rhGH exhibits complexity, and the serum concentration-time curves of YPEG-rhGH in elderly subjects and healthy subjects was observed (Figures 2A,B). After the initial dose, the median time to peak concentration (T_max_) was approximately 46.16 h, which decreased to 22.71 h. However, the T_max_ remained slower compared to younger subjects. The accumulation ratios for C_max_ (RC_max_), C_min_ (RC_min_), and AUC (RAUC) were 1.34, 1.35, and 1.23, respectively. These values suggest a moderate increase in serum exposure of YPEG-rhGH after multiple doses, but no significant drug accumulation in the body. Additionally, the RC_min_ before the week 23 dose was 1.35, consistent with the 13th week results, indicating that steady-state pharmacokinetics were achieved by week 13 of continuous dosing. Elderly subjects receiving 30 μg/kg showed significantly lower serum concentrations than healthy adults at the same dose, with most values comparable to healthy adults receiving 10 μg/kg (Figure 2C). Similar trends were observed for C_max_ and AUC_0-last_, where 30 μg/kg in elderly subjects reached only 7.75% and 19.13% of healthy adult values, respectively, demonstrating markedly reduced absorption in the elderly population (Figure 2D). Gender-stratified analysis at 30 μg/kg revealed higher blood concentrations in younger males compared to elder females (Figure 2E).

#### 3.2.1 PopPK modeling

The PopPK analysis included data from both elderly subjects and healthy adults. According to the experience of quantitative pharmacology in previous studies, combined with the observed data and model results, the time change of serum YPEG-rhGH concentration after abdominal subcutaneous injection of YPEG-rhGH was finally described by a two-compartment model with first-order absorption and nonlinear elimination as a structural model.

The correlation between covariates and base model parameters, as well as among covariates, was assessed using graphical methods. According to the covariate modeling method, when AGE was added to V_max_, OFV decreased by 35.53, when WEIGHT was added to V_max_, OFV decreased by 28.07, when AGE was added to K_a_ and V_1_, OFV decreased by 14.56 and 12.98, respectively. Therefore, the above covariates that significantly affected the PK characteristics of YPEG-rhGH were included, and the process of backward elimination did not find that the covariates could significantly reduce the objective function value, so it was determined as the final model. Table 3 shows the population parameter estimates for the final model. The relative standard error (RSE) of all parameters fitted by the model was less than 30%, indicating that the model parameter estimation was relatively accurate, but it may come from two different age groups, the variation of IIV was large.

**TABLE 3:** Parameters of the YPEG-rhGH PopPK model.

Parameter	Unit	Typical value (RSE%)	IIV (RSE%), %	95% CI	Shrinkage, %
*K*_*a*_	1/h	0.01086 (8.49)	54.77	0.009047–0.01267	22.77
V_1_/F	L	2.387 (15.54)	97.08 (24.30)	1.659–3.115	12.31
V_2_/F	L	21.42 (20.48)	—	12.81–30.04	—
K_m_	μg/L	70.08 (13.78)	54.77	51.13–89.03	0.13
V_max_	μg/h	80.13 (7.82)	—	67.83–92.44	—
CL_2_/F	L/h	0.05575 (10.15)	—	0.04464–0.06686	—
WEIGHT on V_max_	—	2.202 (18.05)	—	1.422–2.982	—
AGE on K_a_	—	−0.6957 (28.49)	—	−1.085–0.3066	—
AGE on V_1_/F	—	1.470 (24.69)	—	0.7574–2.182	—
AGE on V_max_	—	0.7989 (18.37)	—	0.5109–1.087	—
Residual error σ (prop), %	—	28.94 (2.88)	—	0.2730–0.3057	—

### 3.3 Pharmacodynamics

A total of 980 measurements of IGF-1 serum concentrations were provided by 52 subjects without missing IGF-1 data. All IGF-1 data were used to establish the PK/PD model.

**FIGURE 3:**
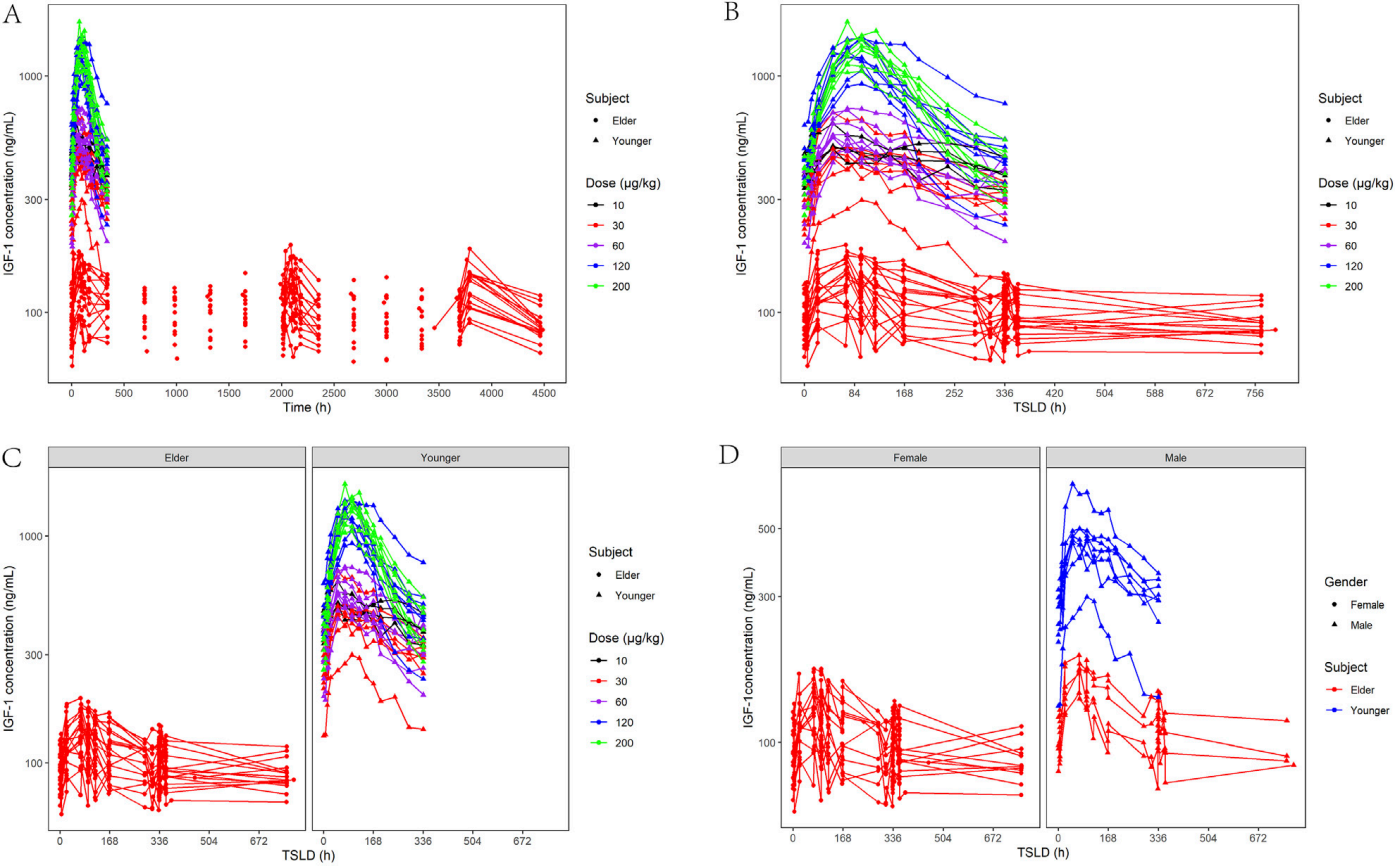
Characterization of IGF-1-based PD after administration of YPEG-rhGH in adult healthy and elderly subjects. **(A,B)** Serum IGF-1 concentration-time curve of subjects after different doses of YPEG-rhGH (semi-log coordinate plot). **(C)** Age-stratified plot of serum IGF-1 concentration and time after administration of YPEG-rhGH (semi-log plot). **(D)** Gender stratification of IGF-1 concentrations and time after YPEG-rhGH administration (semi-log coordinate plot).

Figure 3 shows the changes in serum IGF-1 levels following YPEG-rhGH administration. After receiving a single dose of YPEG-rhGH, the serum IGF-1 level showed a trend of first increasing and then decreasing. The increasing trend of IGF-1 level after multiple doses administration in elderly subjects was similar to that of single dose, and the IGF-1 level fluctuated greatly during the interval of administration. The peak time of IGF-1 level (T_Emax_) was significantly longer than the T_max_ of YPEG-rhGH, indicating a pronounced lag effect in the changes of IGF-1 *in vivo*. After 13 weeks of multiple dosing, C_max_, C_min_, and AUEC remained comparable to week 1 values, and the ratios rC_max_, rC_min_ and rAUEC were 0.99 (17.26%), 1.01 (15.21%) and 0.94 (12.64%), respectively. These findings indicate that IGF-1 levels did not show significant elevation under the Q2W dosing regimen compared to YPEG-rhGH. Week 23 IGF-1 levels showed a slight increase compared to week 13, potentially reflecting delayed steady-state achievement and PD parameter variability due to age-related physiological and pathological factors. Figure 3C demonstrates that elderly subjects receiving 30 μg/kg YPEG-rhGH had absolute levels of IGF-1 that were lower than those in healthy adults in all dose groups, likely attributable to a lower response in elderly subjects and a significantly lower baseline IGF-1 in the elderly population. Age significantly affected IGF-1 levels, but the trend of IGF-1 levels was similar between male and female elderly subjects (Figure 3D).

#### 3.3.1 PK/PD modeling

**FIGURE 4:**
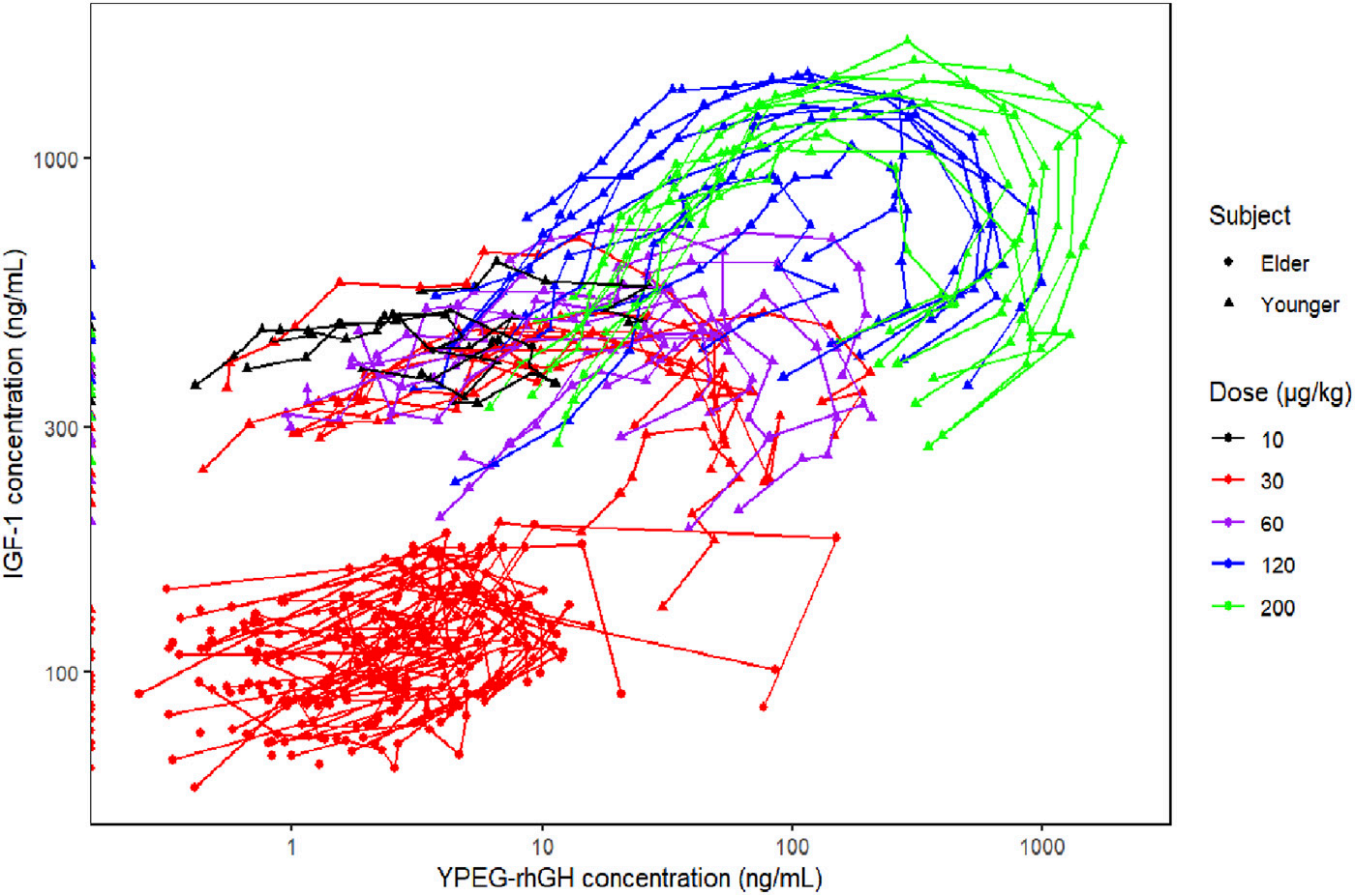
Relationship between YPEG-rhGH serum concentration (PK) and IGF-1 serum concentration (PD).

This study explored the relationship between YPEG-rhGH serum concentration (PK) and IGF-1 serum concentration (PD), revealing a characteristic hysteresis loop between YPEG-rhGH and IGF-1 concentrations (Figure 4). Significant differences were observed in IGF-1 baseline values and trends between elderly and healthy adult subjects. Therefore, the IGF-1 baseline ratio was used as the PD metric in the PK/PD model, while individual PK parameters were extracted from the final PopPK model. The IDR model describing YPEG-rhGH-induced IGF-1 generation was adopted, and a PK/PD model was established in elderly subjects to describe the dose-response relationship.

The basic model fully estimated the random effects of E_max_, EC_50_, K_in_, K_out_, γ and other parameters, but the fitting was poor. After optimization, when γ was fixed at 1, and only additive random effects for EC_50_ and K_out_ were retained, the parameter estimation was more reasonable, the model fitted well, and the condition number was significantly reduced, so the final basic model was established. Population parameter estimates for the final model for older subjects are shown in Table 4.

**TABLE 4:** PK/PD final model parameters of IGF-1 relative to baseline ratio in elderly subjects.

Parameter	Unit	Typical value (RSE%)	IIV (RSE%), %	95% CI	Shrinkage, %
K_in_	ng/(mL·h)	0.023 (14.79)	—	0.01615–0.02938	—
K_out_	1/h	0.023 (15.50)	10.27 (3.901)	0.01603–0.03006	6.74
E_max_	—	2.245 (21.80)	—	1.283–3.207	—
EC_50_	ng/mL	50.74 (37.68)	78.76 (30.66)	13.16–88.32	9.97
Residual error, σ(add), ng/mL	—	13.20 (6.13)	—	0.1161–0.1479	—

### 3.4 Model evaluation

**FIGURE 5:**
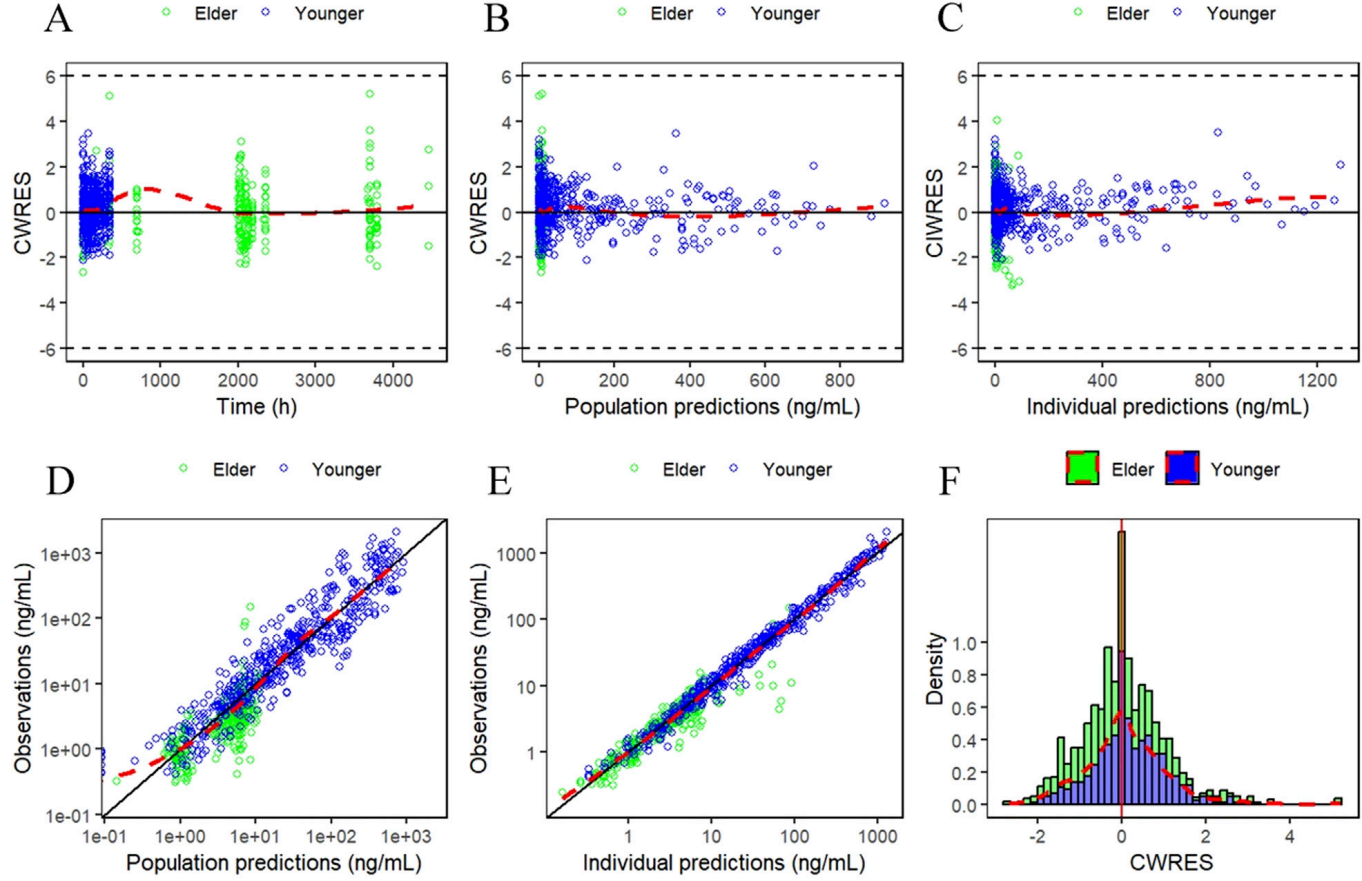
Goodness-of-fit (GOF) plots for the final PopPK model. **(A)** Conditionally weighted residuals plotted against time after first dose. **(B and C)** Conditionally weighted residuals plotted against population and individual predictions of YPEG-rhGH concentration. **(D and E)** YPEG-rhGH concentration observations plotted against population predicted values, individual predicted values. **(F)** Histogram of conditional weighted residual probability density. The red dashed line is the fitted trend line.

**FIGURE 6:**
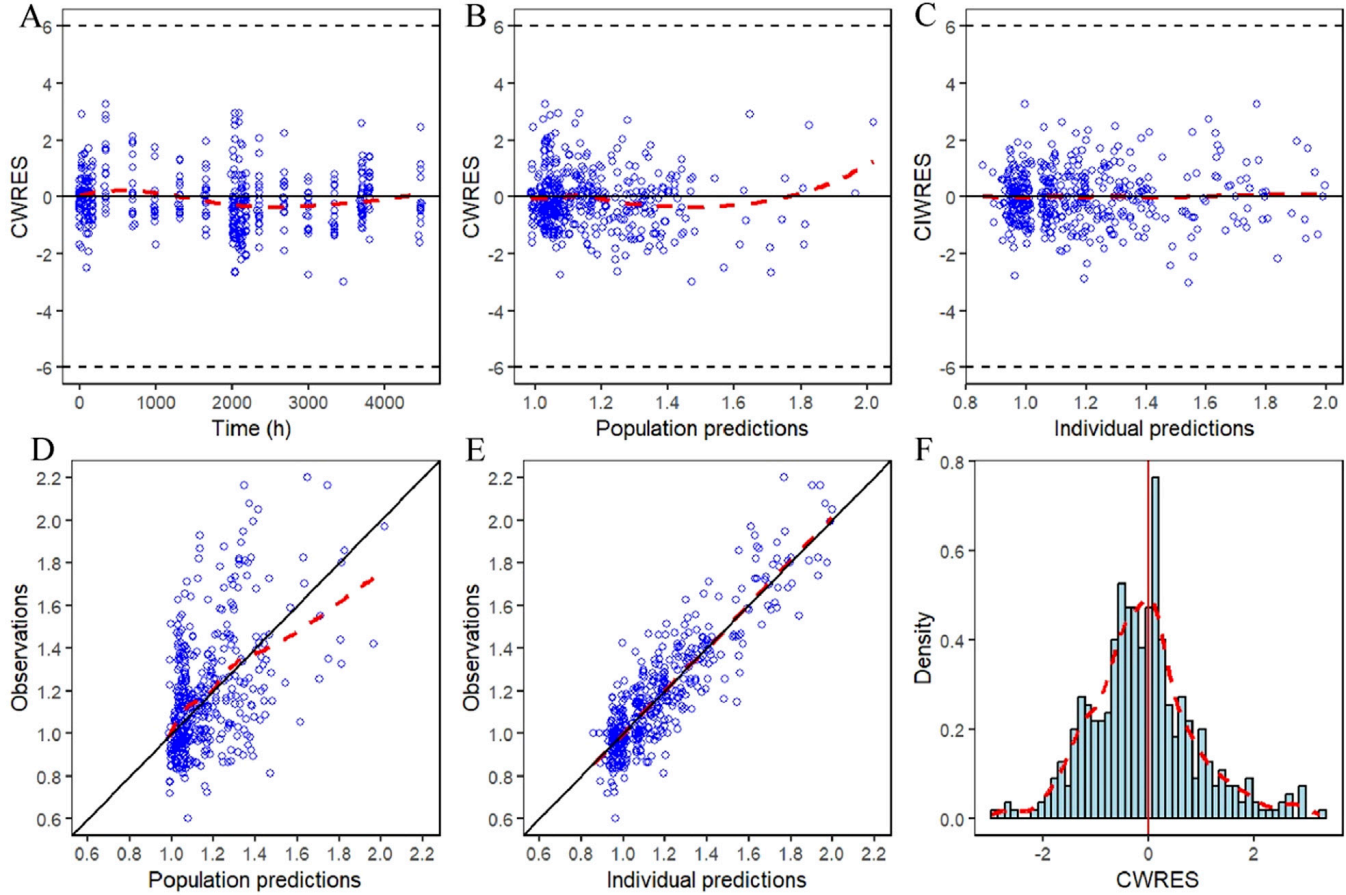
IDR model GOF plot of relative baseline ratios of IGF-1 in elderly subjects given YPEG-rhGH. **(A)** Conditionally weighted residuals plotted against time after first dose. **(B,C)** Conditional weighted residuals plotted the group and individual predicted values of IGF-1 relative to baseline ratios in older subjects. **(D,E)** Plotting of observed IGF-1 relative baseline ratios against group predicted values, individual predicted values in elderly subjects. **(F)** Histogram of conditional weighted residual probability density. The red dashed line is the fitted trend line.

The GOF plots for the final PopPK/PD model in Figures 5, 6 demonstrated good agreement between observations and predictions with no evidence of structural misspecification. The success rates for both the PopPK and PD model fittings were 100%.

The median and 95% CI from the bootstrap results closely aligned with the parameter estimates and their 95% CIs. The typical values of the final model parameters all fell within the bootstrap 95% CIs, indicating good precision in parameter estimation. Supplementary Tables S2 and S3 provide the data support for this section.

**FIGURE 7:**
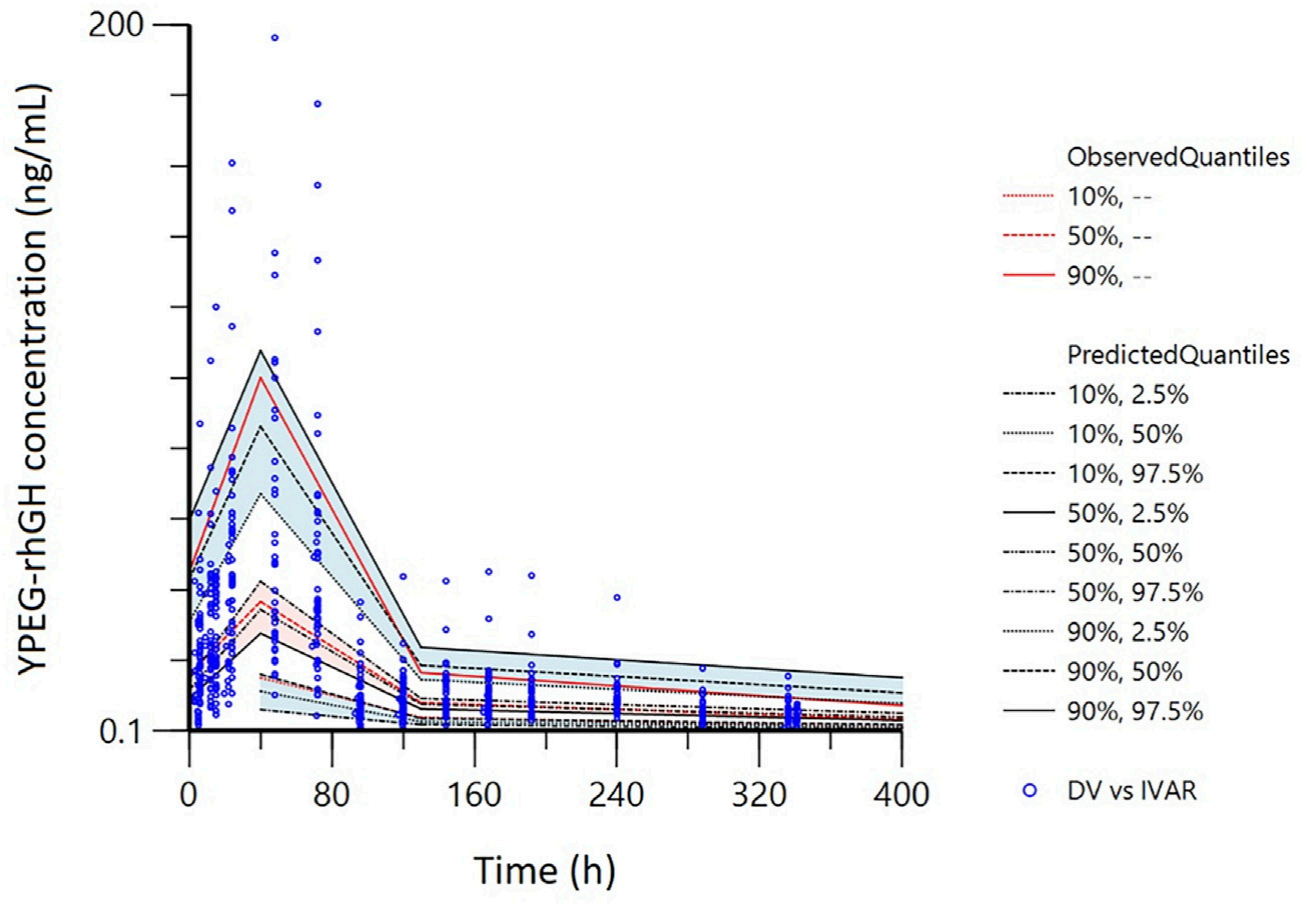
Prediction- and variability-corrected visual predictive checks (pvc-VPC) for the final PopPK model. The hollow points are the observed plasma concentrations of YPEG-rhGH. The shaded area is the 95%CI of each line corresponding to the predicted percentile.

**FIGURE 8:**
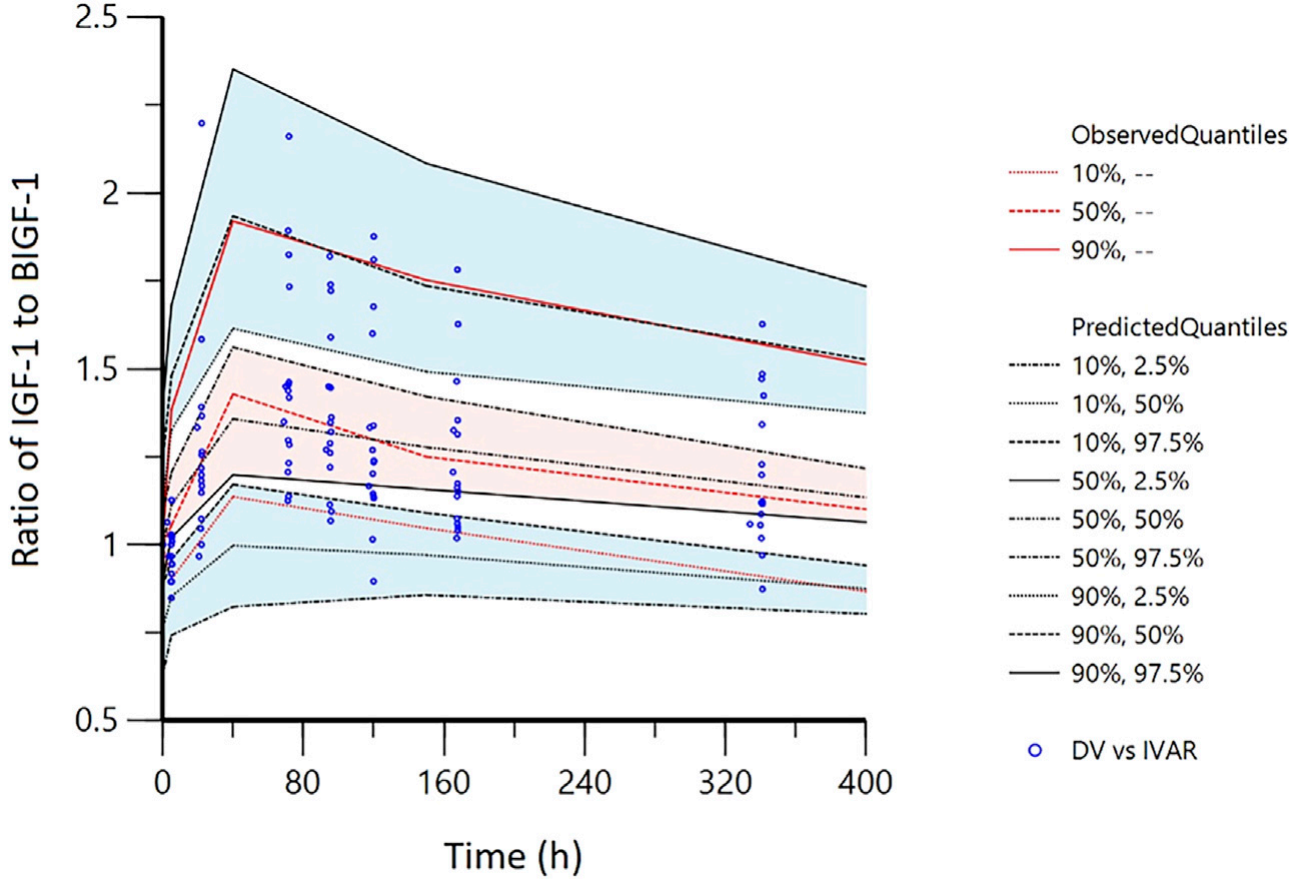
Visual predictive checks (VPC) of IDR model of IGF-1 relative to baseline ratio after YPEG-rhGH administration in elderly subjects. The hollow points are the ratio of the observed value of serum IGF-1 concentration to the baseline: the three lines from the bottom to the top are 10%, median and 90% quantile of the observed data, respectively. The shaded area is the 95% CI of each line corresponding to the predicted percentile.

The pvcVPC plots in Figures 7, 8 showed that the 10th, 50th, and 90th percentiles of the observed data fell entirely within the 95% confidence intervals of simulations, supporting the model’s robust predictive performance.

### 3.5 Simulations

**FIGURE 9:**
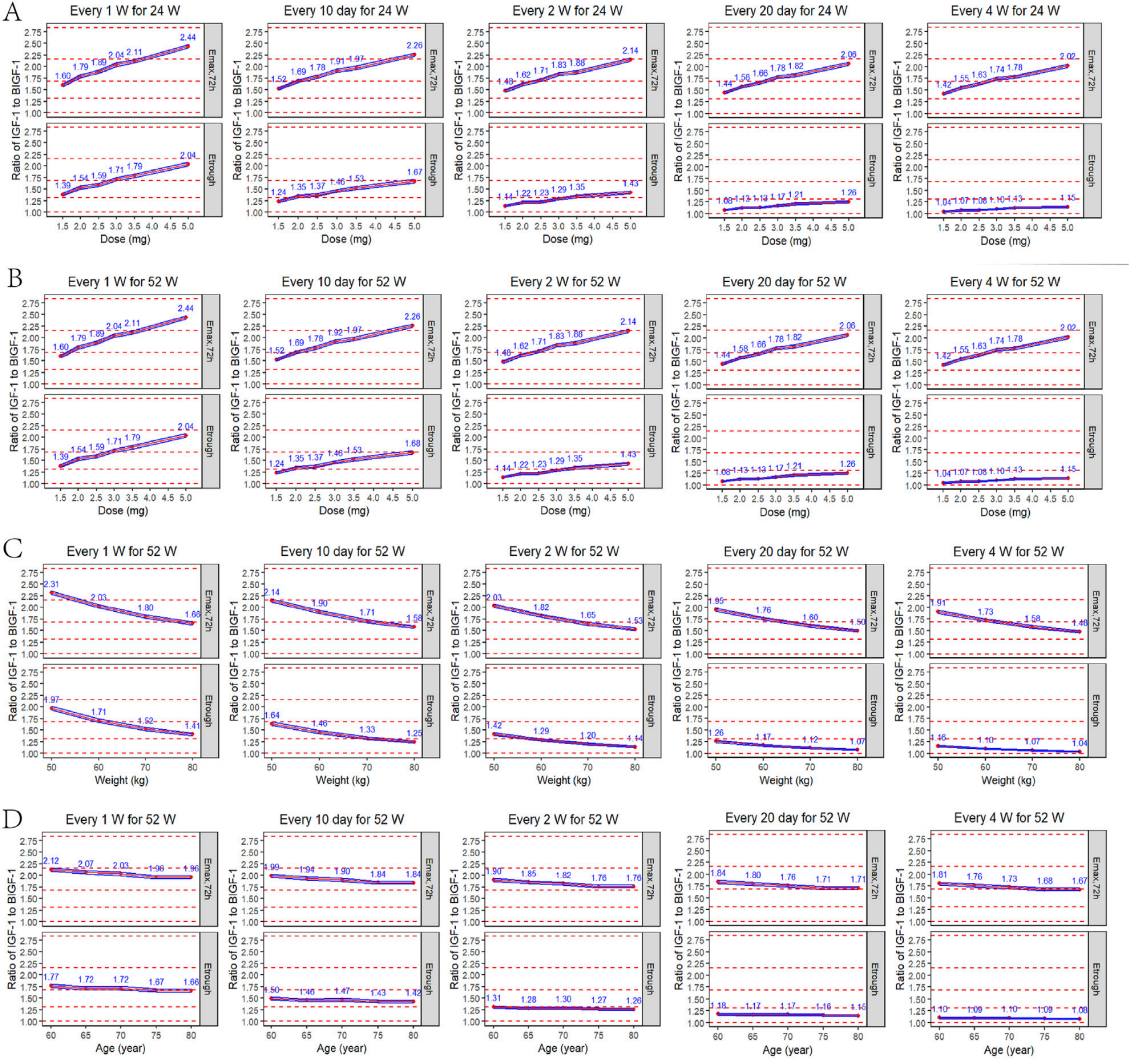
Peak and trough IGF-1 relative baseline ratios after the last dose of each regimen. **(A)** Simulated IGF-1 relative baseline ratio in elderly subjects after 24 weeks of sequential administration at different doses/intervals (n = 1,000). **(B)** Simulated IGF-1 relative baseline ratio in elderly subjects after 52 weeks of sequential administration at different doses/intervals (n = 1,000). **(C)** Simulated results of IGF-1 relative baseline ratios after 52 consecutive weeks of administration (formulation size: 3 mg) in elderly subjects (70 years) of different body weights (50, 60, 70, 80 kg) (n = 1,000). **(D)** Simulated results of IGF-1 relative baseline ratios after 52 consecutive weeks of administration (formulation: 3 mg) in elderly subjects (60 kg) of different ages (60, 65, 70, 75, 80 years) (n = 1,000). E_max,72h_: peak IGF-1 relative baseline after the last dose; E_trough_: trough after the last dose; blue line interval: 95% CI for the mean IGF-1 relative baseline ratio; red dashed line: reference line for the relative baseline ratio from bottom to top for the increase in the IGF-1 level by 0, 1, 2, 3 and 4 SD.

**FIGURE 10:**
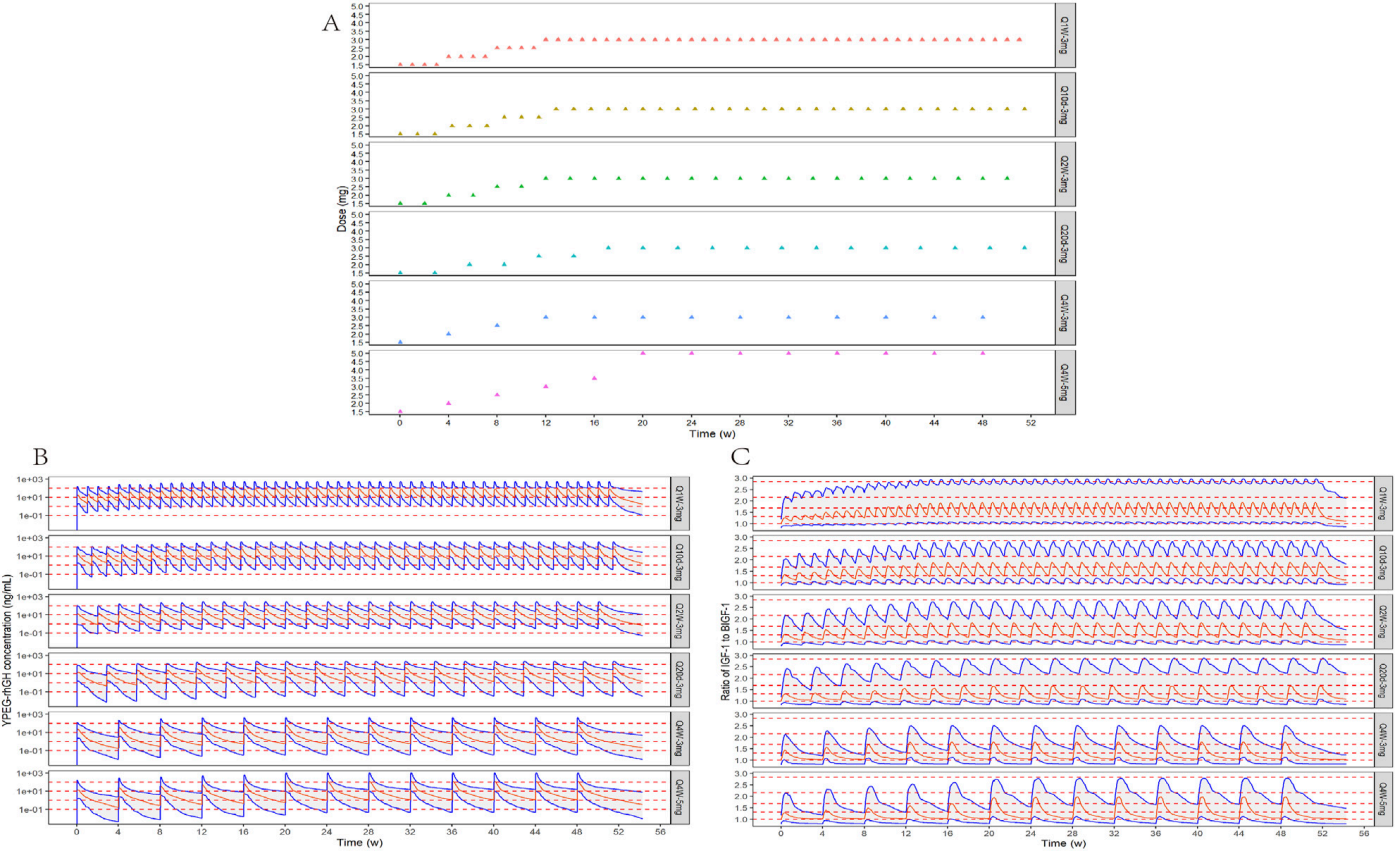
Titration schemes and simulation results. **(A)** Schematic diagram of six different simulated titration protocols for 52 consecutive weeks. **(B)** Simulation of YPEG-rhGH blood concentration-time curves for different titration schemes (n = 300, semi-logarithmic coordinate plots). The red solid line is the median YPEG-rhGH blood concentration curve, the blue solid line is the 5% and 95% quantile line from bottom to top, and the red dashed line is the blood concentration 0.1, 1, 10, and 100 ng/mL reference line from bottom to top. **(C)** Simulation of IGF-1 relative baseline ratio-time curves after administration of different titration regimens. The red solid line is the median IGF-1 relative baseline ratio curve, the blue solid line is the 5% and 95% quantile line from bottom to top, and the red dashed line is the reference line of relative baseline ratios corresponding to 0, 1, 2, 3, and 4 SD increase in IGF-1 levels in 70-year-olds from bottom to top.

According to the parameters of the final PopPK/PD model in elderly subjects, the relative baseline ratio of IGF-1, an efficacy indicator, after different dosing regimens and different dosing intervals in elderly patients, was simulated. The peak and trough data of the relative baseline ratio of IGF-1 after the last administration of each regimen are shown in Figure 9. IGF-1 peak and trough values remained consistent between 24 and 52 weeks, demonstrating PD steady-state achievement following 12 YPEG-rhGH doses. While dosing intervals exerted minimal influence on peak values, they significantly affected trough concentrations. Simulations of 3 mg/dose in elderly subjects of different body weights showed a significant decrease in the ratio of IGF-1 relative to baseline with increasing body weight. The effect of different ages on the ratio of baseline IGF-1 increase by drugs also showed a downward trend with the increase of age, but the effect on the dosing regimens with longer dosing intervals was less. Titration scheme analyses demonstrated that PK reached steady state faster than PD (Figure 10). Quantile shading (5% and 95% intervals) also shows large differences between elderly individuals. Future clinical trials should consider implementing optimized dosing regimens tailored to target IGF-1 trough level increases.

### 3.6 Safety and tolerability

A total of 16 subjects were included for safety analysis. During the study, 15 of the 16 subjects (93.8%) experienced a total of 44 AEs, most of which were grade 1, only 2 of which were grade 2, one subject (6.3%) had gastrointestinal diseases/non-infectious gingivitis, and one subject (6.3%) had kidney and urinary system diseases/urinary calculi, which were not related to the test drug. No grade 3 or higher treatment emergent adverse events (TEAEs) were observed. 14 subjects (87.5%) had 28 adverse events related to the trial drug, all of which were grade 1 in severity. All of them resolved spontaneously or returned to clinically acceptable levels without intervention. No serious, fatal, or other significant AEs were observed throughout the study, and no subjects discontinued test drugs or withdrew due to AEs.

Among subjects who experienced at least one TEAE related to the trial drug, the proportion of subjects with examination-related TEAE was 81.3%, primarily in the form of elevated blood insulin (31.3%), elevated blood cholesterol (25.0%), elevated blood triglycerides (12.5%), and elevated blood pressure (6.3%). The proportion of subjects experiencing gastrointestinal disorders (diarrhea) was 12.5%, and the proportion of subjects experiencing systemic diseases and various reactions at the injection site (injection site pain) was 6.3%. All other adverse events related to the trial drug occurred only once.

## 4 Discussion

YPEG-rhGH is a novel recombinant human growth hormone that utilizes highly biologically active, non-N-terminal site-specific modifications to enhance the protection of rhGH, aiming to reduce the required dosage while maintaining efficacy and improving long-term medication safety. This study employed a two-compartment model with nonlinear elimination to quantify the PK characteristics of serum YPEG-rhGH following subcutaneous injection in elderly subjects and healthy adult. The IDR model of YPEG-rhGH-induced IGF-1 generation was used to describe the time-dependent changes in IGF-1 levels after YPEG-rhGH administration. The study focused on investigating the influence of subject-related factors on the PK/PD parameters of YPEG-rhGH serum concentrations and IGF-1 levels following YPEG-rhGH administration.

The PK/PD analysis of this study showed that YPEG-rhGH exhibited nonlinear elimination characteristics after subcutaneous injection into the abdomen of healthy adult subjects in the dose range of 10–200 μg/kg, presenting a typical biomolecule PK profile. Notably, PK parameters after YPEG-rhGH injection in the elderly differed from those of healthy adult subjects, with significantly lower levels of YPEG-rhGH in elderly subjects than in healthy adult subjects, but also two cases of elderly male subjects with levels comparable to those of healthy adults at the same dose, and with distributional characteristics consistent with a two-compartment model. At the administered dose of YPEG-rhGH of 30 μg/kg, the overall plasma concentration exposure of elderly subjects was significantly lower than that of healthy adult subjects, this discrepancy may be related to the significantly lower absorption of YPEG-rhGH in elderly subjects. It has been established that many specific morphological, physiological, cognitive and pathological changes occur with increasing age, resulting in decreased absorption efficiency in the elderly population, decreased blood flow to subcutaneous tissues, decreased capillary density, weakened skin barrier function, and decreased skin elasticity may all affect drug absorption (Perrie et al., 2012; Khan and Roberts, 2018). In addition, there was a higher proportion of females among the elderly subjects, which may be one of the key factors affecting the absorption efficiency of YPEG-rhGH at the site of administration, taking into account the gender differences in subcutaneous adipose tissue. Gender differences in adipose tissue are mainly reflected in distribution and metabolic activity. Females usually have a higher percentage of subcutaneous fat, whereas males tend to have a greater distribution of visceral fat (Karastergiou et al., 2012). And this difference affects drug absorption further affecting local metabolism and distribution, so the different subcutaneous adipose tissue may be an important reason for affecting the absorption of the subcutaneous formulation of YPEG-rhGH at the site of administration.

In this study, during the process of PopPK modeling, it was found that the PopPK model based on the elderly population alone was not stable due to the limited number of cases of elderly subjects. In order to improve the reliability of the model, the study included the data of healthy adult subjects in the analysis, and finally a stable PopPK model was established. Through covariate analysis, AGE and WEIGHT were identified as significant covariates affecting the pharmacokinetics of YPEG-rhGH, and these factors explained, to some extent, the PopPK differences between healthy adults and elderly subjects; specifically, AGE showed a significant effect on the key pharmacokinetic parameters of K_a_, V_1_/F, and V_max_, whereas WEIGHT significantly affected V_max_. After administration of YPEG-rhGH, changes in IGF-1 levels over time lagged behind changes in YPEG-rhGH concentrations over time; therefore, an IDR model structure was used for this PopPK/PD analysis. The median T_Emax_ of peak IGF-1 levels in different dose groups and subjects ranged from 72 to 96 h. In healthy adults, there was a tendency for T_Emax_ to be delayed with increasing dose; the median T_Emax_ in healthy adult subjects and elderly subjects at the same dose was similar, but the range of T_Emax_ in elderly subjects was 22.22–341.76 h, suggesting that elderly subjects greater inter-individual variability.

Although this study preliminarily revealed the effect of age on the pharmacokinetics of YPEG-rhGH, it is noteworthy that the PK/PD model constructed based on PK and IGF-1 data from 16 elderly subjects after administration of YPEG-rhGH also revealed in the simulation results of the elderly that different ages (60–80 years) had less effect on both the concentration level of YPEG-rhGH and the pharmacodynamic index IGF-1 levels were both smaller. This phenomenon may be attributed to the limited sample size of elderly subjects or the substantial inter-individual variability in the elderly population, which aligns with the previously observed extensive inter-individual variability in this population and has been reported in the literature (Mangoni et al., 2013; Maher et al., 2021).

In this study, YPEG-rhGH also proved to be safe and well-tolerated, with no serious, fatal or other significant adverse events reported. All AEs were grade 1 or 2 in severity, and no subjects discontinued treatment or withdrew, consistent with previous findings (Guan et al., 2018). Among the drug-related AEs, the incidence of elevated blood insulin was 31.3%. Previous studies indicate that GH replacement therapy, particularly during initial phases and at higher doses, may transiently increase blood glucose levels and insulin resistance. However, a meta-analysis showed that short-term (6–12 months) GH therapy affects glucose metabolism in adult GHD patients, and the negative effects of glucose homeostatic parameters were not significant at longer durations (more than 12 months). This suggests that the beneficial effects of GH on body composition and fitness may occur after longer periods of treatment and eventually outweigh the negative effects of GH on glucose metabolism, resulting in a return of glucose parameters to baseline values. In our study, which was administered for a total of 23 weeks, we observed increased blood insulin in five cases, but fasting blood glucose and glycosylated hemoglobin levels remained essentially within normal ranges, consistent with the literature (Biller et al., 2011; Zhou et al., 2021). Other studies have shown that children or adults with GHD have abnormal lipid metabolism, with higher levels of LDL and triglycerides than normal controls of the same age, and that cholesterol and LDL levels decrease after GH treatment, with no change in triglycerides (McConnell et al., 2001; Monson, 2003). In this study, we observed transient mild elevation of blood cholesterol, blood triglycerides, and LDL in some elderly healthy subjects with the use of this product and recovered on their own, which is inconsistent with previous studies and may be affected by age, BMI, GH treatment conditions, and the use of lipid-modulating drugs. Given the small sample size, the differences in drug response between elderly subjects and healthy individuals and patients need to be further explored. Previous studies have shown specific adverse effects occurring during growth hormone replacement therapy in adults with GHD that are primarily related to sodium retention, including edema, arthralgia, myalgia, paresthesia, and carpal tunnel syndrome. Elderly, overweight, and female patients appear to be at higher risk for these adverse effects, which were not observed in this study (Kargi and Merriam, 2013). While some studies report injection-site fat atrophy with PEGylated GH, this was not observed in our study (Rasmussen et al., 2010; Miller et al., 2020).

However, there are limitations to this study: as noted above, the elderly cohort in this study primarily consisted of healthy older adults, which may lead to notable differences in health status compared to the general elderly patient population. Due to the relatively stringent inclusion criteria (e.g., blood pressure below 140/90 mmHg, 18 kg/m^2^ ≤BMI ≤30 kg/m^2^, *etc.*), our findings may be more applicable specifically to healthy older individuals. Extrapolation of the model to other age groups or populations with different physiological or pathological conditions requires further external validation to confirm its predictive reliability and robustness. Future studies should aim to expand the sample size and include a broader range of health conditions to enhance the generalizability of the results. The small number of subjects limits the statistical power of the analyses, including covariate analyses, and therefore, some covariates that were not identified as significant in this analysis may have been missed. For example, in these two projects, only the elderly subjects project included females. Given the skewed distribution of the male-to-female gender ratio, and in the subsequent multivariate covariate analysis, we found that gender was significantly correlated with several key covariates such as AGE, WEIGHT, and biochemical markers. Therefore, we decided to prioritize retaining the more direct and clinically significant covariates. As a result, gender was not included in the population pharmacokinetic modeling analysis of this study. The potential influence of gender covariates on PK/PD modeling will be further evaluated pending subsequent collection of additional trial data, after more elderly subjects are included in the Phase II and Phase III clinical trials. In the meantime, the inclusion of more data from elderly subjects will also help us to further and more comprehensively validate the effect of AGE covariates on PopPK/PD in the elderly population.

## 5 Conclusion

This study confirmed that the pharmacokinetic characteristics of YPEG-rhGH in healthy adults and elderly subjects can be well described using a two-compartment model with first-order absorption and nonlinear elimination. The final model identified AGE as a significant covariate for K_a_, V_1_/F, and V_max_, while WEIGHT was a significant covariate for V_max_. Specifically, as AGE increases, K_a_ decreases, V_1_/F, V_max_ increases, and *in vivo* drug exposure tends to decrease; as WEIGHT increases, V_max_ increases, which also results in decreased drug exposure *in vivo*. In terms of pharmacodynamics, the study successfully established a PK/PD model applicable to the elderly population using the IDR model of IGF-1 relative to the baseline ratio. Based on the simulation results, the baseline ratio of administration could rise to the range of 1.14–1.43 after a 1.5–5 mg dose of 24W/52W was given at Q2W in the elderly, with the 3 mg dose elevating the ratio by approximately 1 SD. Both AGE and WEIGHT could affect the level of IGF-1 trough concentration. Overall, subjects were safe and well tolerated after receiving YPEG-rhGH injections.

## Data Availability

The original contributions presented in the study are included in the article/Supplementary Material, further inquiries can be directed to the corresponding authors.
