# Supplementary material for: Population pharmacokinetics/pharmacodynamics and safety of YPEG-rhGH in elderly subjects
Source: Front Pharmacol. 2025 Nov 25;16:1651323. doi: 10.3389/fphar.2025.1651323 (PMC12685806; doi:10.3389/fphar.2025.1651323)
Supplement: Supplementary file 1 [file DataSheet1.docx]

**Supplementary Table 1.** Comparison table between PopPK final model and Bootstrap parameters

|  | Final model | | Bootstrap* | |
| --- | --- | --- | --- | --- |
|  | Estimates(RSE%) | 95%CI | Estimates(RSE%) | 95%CI |
| PK parameters | | | | |
| K_a_, 1/h | 0.01086(8.49) | 0.009047~0.01267 | 0.01102 | 0.009160~0.01403 |
| V_1_/F, L | 2.387(15.54) | 1.6591~3.1155 | 2.431 | 1.734~3.441 |
| V_2_/F, L | 21.42(20.48) | 12.81~30.04 | 20.83 | 11.15~43.07 |
| K_m_, μg/L | 70.08(13.78) | 51.13~89.03 | 71.45 | 45.26~106.7 |
| V_max_, μg/h | 80.13(7.82) | 67.83~92.44 | 80.84 | 63.33~107.7 |
| CL_2_/F, L/h | 0.05575(10.15) | 0.04464~0.06686 | 0.06058 | 0.02881~0.09989 |
| WEIGHT on V_max_ | 2.202(18.05) | 1.422~2.982 | 2.194 | 0.6494~3.461 |
| AGE on K_a_ | -0.6957 (28.49) | -1.085~-0.3066 | -0.7091 | -0.9709~-0.4449 |
| AGE on V_1_/F | 1.470 (24.69) | 0.7574~2.182 | 1.398 | 0.5416~2.275 |
| AGE on V_max_ | 0.7989 (18.37) | 0.5109~1.087 | 0.7819 | 0.3734~1.186 |
| Inter-individual | | | | |
| ω(V_1_/F), % | 97.08(24.30) | 50.84~143.3 | 94.35(26.82) | 44.75~143.9 |
| ω(K_a_), % | 54.77 | - | 54.77 | - |
| ω(K_m_), % | 54.77 | - | 54.77 | - |
| Residual error | | | | |
| σ (prop), % | 28.94 (2.88) | 27.30~30.57 | 28.61 | 24.65~32.28 |

*OFV：4722.9

## Supplementary Table 2. Comparison table between the final PD model and Bootstrap parameters for the elderly

|  | PD model for the elderly | | Bootstrap* | |
| --- | --- | --- | --- | --- |
|  | Estimates (RSE%) | 95%CI | Estimates | 95%CI |
| PK parameters | | | | |
| K_in_, ng/(mL·h) | 0.023(14.79) | 0.01615~0.02938 | 0.02347 | 0.01824~0.03798 |
| K_out_, 1/h | 0.023(15.50) | 0.01603~0.03006 | 0.02395 | 0.01809~0.03935 |
| E_max_ | 2.245(21.80) | 1.283~3.207 | 2.146 | 1.213~3.092 |
| EC_50_, ng/mL | 50.74(37.68) | 13.16~88.32 | 46.14 | 19.86~83.51 |
| Inter-individual | | | | |
| ω(EC_50_), % | 78.76(30.66) | 31.43~126.1 | 81.95(45.38) | 9.055~154.8 |
| ω(K_out_), % | 10.27(3.901) | 9.485~11.06 | 9.549(4.827) | 8.646~10.45 |
| Residual error | | | | |
| σ(add), ng/mL | 13.20(6.13) | 11.61~14.79 | 13.14 | 11.52~14.64 |

*OFV：-440.5

**Supplementary Table 3.** Clinical studies included in the analysis

| **Study No.** | **Study design** | **Dose regimen** | **Subjects** | **PK sampling design** | **IGF-1 sampling design** |
| --- | --- | --- | --- | --- | --- |
| TB2208GH | Phase I,  single-center,  open and non-randomized  study | 30 μg/kg/2 weeks of YPEG-rhGH injection for 23 consecutive weeks | Healthy elderly subjects,n = 16 | Before (0 h) and 8, 24, 72, 96, 120, 168, 336 h after the first dose; before the third dose; before and 8, 24, 72, 96, 120, 168, 336 h after the seventh dose; before and 8 h and 96 h after the twelfth dose (follow-up until 5 weeks after discontinuation) | Before (0 h) and 8, 24, 72, 96, 120, 168, 336 h after the first dose; before the third, fourth, fifth, and sixth doses; before and 8, 24, 72, 96, 120, 168, and 336 h after the seventh dose; before the ninth, tenth, and eleventh doses; and before and 8 and 96 h after the twelfth dose (follow-up until 5 weeks after discontinuation) |
| TB1010GH | Phase I,  single-center,  open and randomized study | First stage:0.1 IU/kg or 0.15 IU/kg of Saizen® for 7 consecutive days  Second stage: a single dose of 10 μg/kg, 30 μg/kg, 60 μg/kg, 120 μg/kg, or 200 μg/kg of YPEG-rhGH | Healthy male subjects,n = 36 | First stage: 30 min before (0 h) and 6, 12, 15, 24, 48, 72, 96, 120, 144, 168, 192, 240, and 288 h after the first dose.  Second stage: 30 min before the first dose (0 h) and 6, 12, 15, 24, 48, 72, 96, 120, 144, 168, 192, 240, 288, and 336 h after the first dose (follow-up to 12 months) | First stage: 30 min before (0 h) and 6, 12, 15, 24, 48, 72, 96, 120, 144, 168, 192, 240, and 288 h after the first dose.  Second stage: 30 min before the first dose (0 h) and 6, 12, 15, 24, 48, 72, 96, 120, 144, 168, 192, 240, 288, and 336 h after the first dose (follow-up to 12 months) |

**Supplemental table 4. Test of distribution balance for key covariates (age and body weight) between the two subject groups**

| **Independent samples test** | | | | | | | |  |  |  |
| --- | --- | --- | --- | --- | --- | --- | --- | --- | --- | --- |
|  | | **Levene’s test for equality of variances** | | **t-test for equality of means** | | | | | | |
|  |  | **F** | **Sig.** | **t** | **Degree of freedom** | **Sig. (2-tailed)** | **Mean difference** |  | **95% Confidence interval of the difference** | |
|  |  |  |  |  |  |  |  | **Std. error difference** | **Lower** | **Upper** |
| **AGE** | **Equal variances assumed** | **13.086** | **.001** | **-21.123** | **50** | **.000** | **-36.660** | **1.735** | **-40.146** | **-33.174** |
|  | **Equal variances not assumed** |  |  | **-27.459** | **49.925** | **.000** | **-36.660** | **1.335** | **-39.341** | **-33.978** |

| **Independent samples test** | | | | | | |  |  |  |  |
| --- | --- | --- | --- | --- | --- | --- | --- | --- | --- | --- |
|  | | **Levene’s test for equality of variances** | | **t-test for equality of means** | | | | | | |
|  |  | **F** | **Sig.** | **t** | **Degree of freedom** | **Sig. (2-tailed)** |  |  | **95% Confidence interval of the difference** | |
|  |  |  |  |  |  |  | **Mean difference** | **Std. error difference** | **Lower** | **Upper** |
| **WEIGHT** | **Equal variances assumed** | **.380** | **.541** | **2.313** | **50** | **.025** | **4.534** | **1.961** | **.596** | **8.472** |
|  | **Equal variances not assumed** |  |  | **2.164** | **24.910** | **.040** | **4.534** | **2.095** | **.218** | **8.850** |

**Supplemental table 5. Comparison of PopPK/PD model parameter estimates using AGE versus TRIAL as a covariate**

| Full model K_a_-AGE V_1_-AGE V_max_-AGE V_max_-WEIGHT | | | Full model K_a_-TRIAL V_1_-TRIAL V_max_-TRIAL V_max_-WEIGHT | | |
| --- | --- | --- | --- | --- | --- |
|  | | |  | | |
| Parameter | Unit | Typical value(RSE%) | Parameter | Unit | Typical value(RSE%) |
| K_a_ | 1/h | 0.01086(8.49) | K_a_ | 1/h | 0.006540(30.03) |
| V_1_/F | L | 2.387(15.54) | V_1_/F | L | 7.3321(30.05) |
| V_2_/F | L | 21.42(20.48) | V_2_/F | L | 26.62(15.92) |
| K_m_ | μg/L | 70.08(13.78) | K_m_ | μg/L | 98.98(16.01) |
| V_max_ | μg/h | 80.13(7.82) | V_max_ | μg/h | 163.7(73.20) |
| CL_2_/F | L/h | 0.05575(10.15) | CL_2_/F | L/h | 0.06548(6.105) |
| WEIGHT on V_max_ | - | 2.202(18.05) | trial on K_a_ | - | 1.150(66.10) |
| AGE on K_a_ | - | -0.6957(28.49) | trial on V_1_/F | - | -0.7142(15.91) |
| AGE on V_1_/F | - | 1.470(24.69) | trial on V_max_ | - | -0.5849 (60.13) |
| AGE on V_max_ | - | 0.7989(18.37) |  | | |
| Residual error,  σ (prop), % | - | 28.94(2.88) | Residual error,  σ (prop), % | - | 29.17(2.35) |
| OFV | 4722.9265 | | OFV | 4776.9778 | |
| Condition number | 13195.382 | | Condition number | 139986.4 | |

## Supplementary Table 6. The range of baseline values in elderly subjects

| Variable | n | Minimum | Maximum | Range | Mean | Std. dev. | CV% |
| --- | --- | --- | --- | --- | --- | --- | --- |
| IGF-1 | 16 | 64.9 | 126.0 | 61.1 | 93.8 | 17.4 | 20.1 |

**Supplemental Figure 1:** PopPK model individual fit curve (linear coordinate plot) after subjects were given YPEG-rhGH


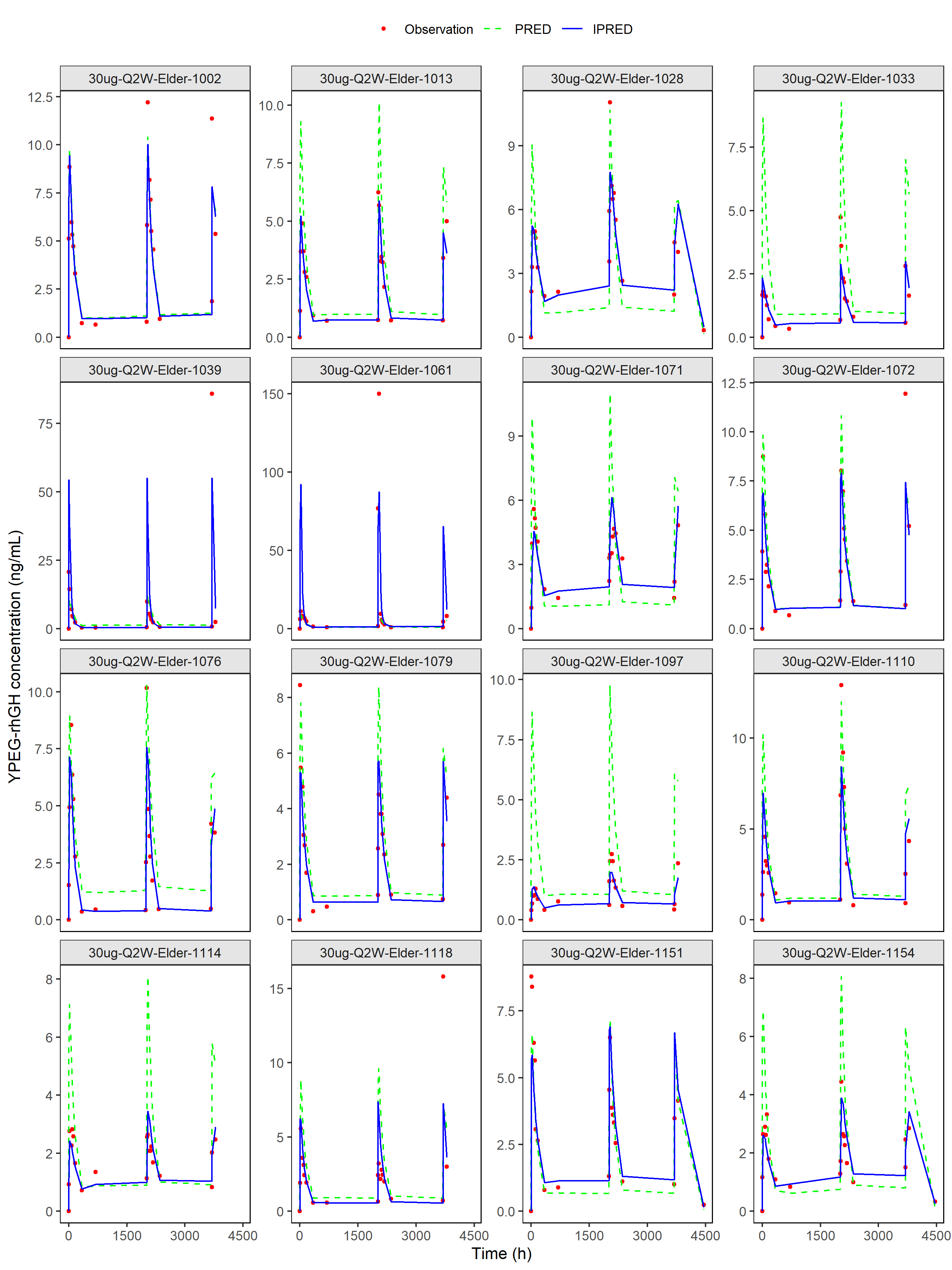


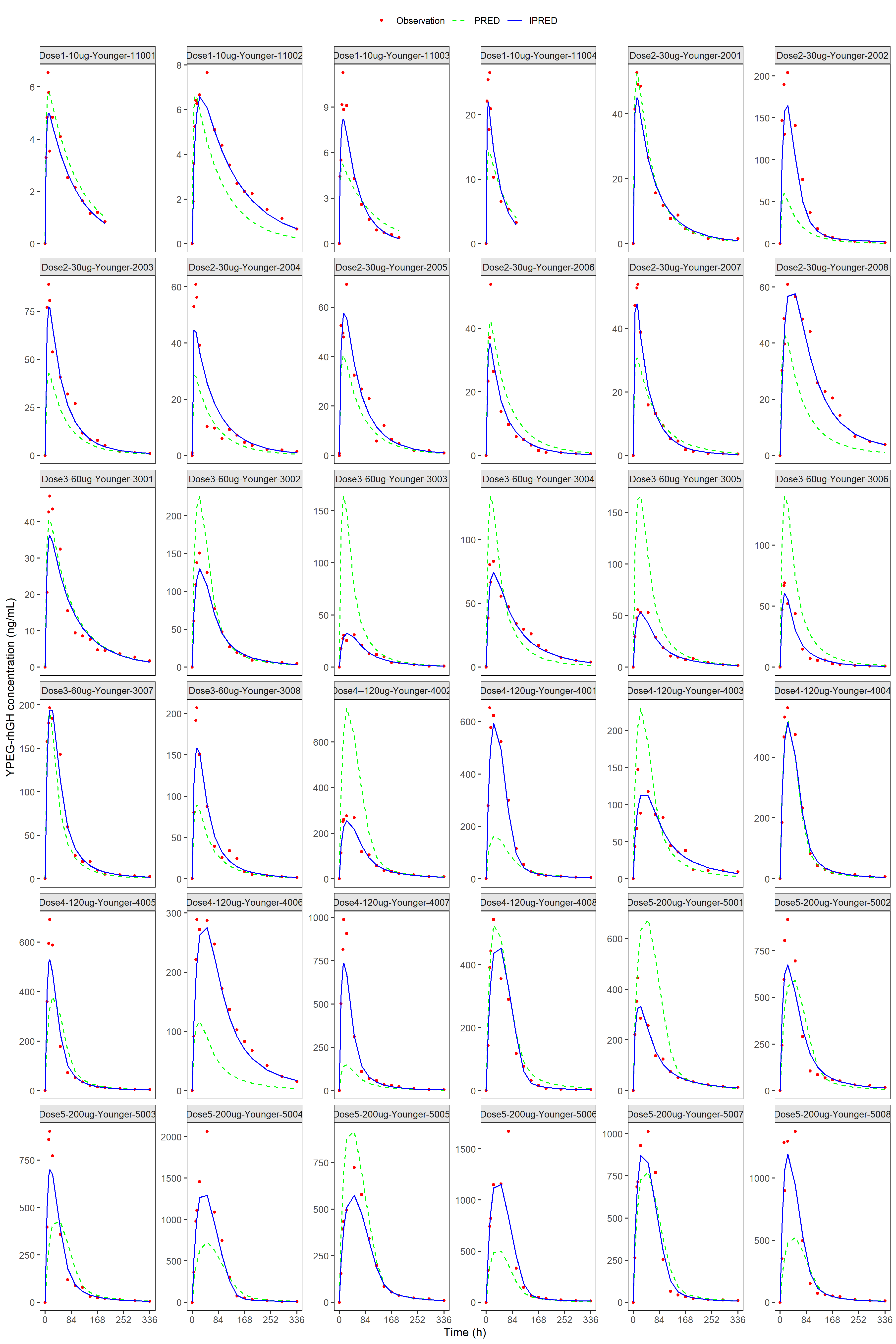


**Supplemental Figure 2：**Individual fitting curves (linear coordinate plots) for PK/PD (IGF-1) model in the elderly subject population


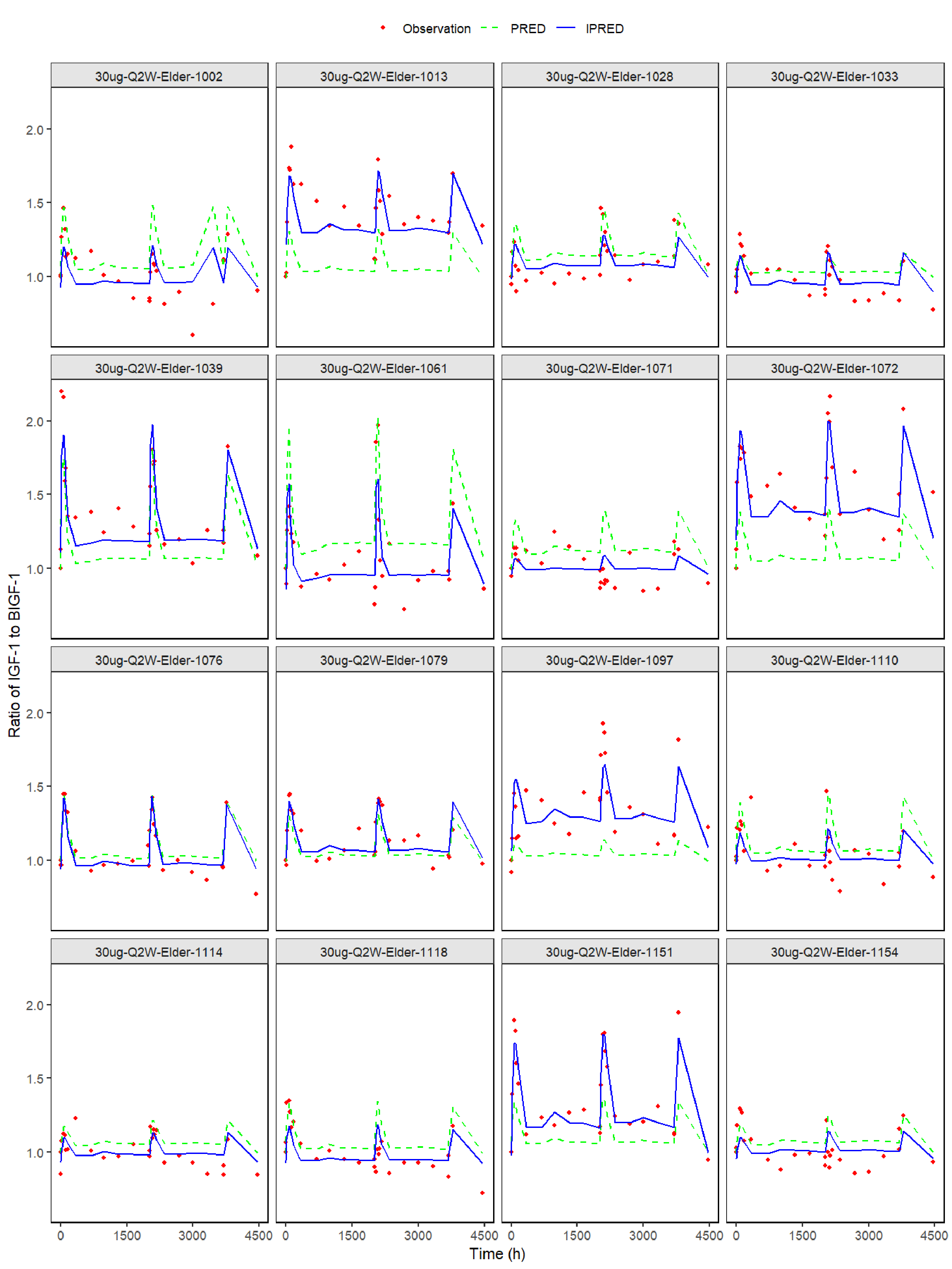


**Supplemental Figure 3:** Diagnostic goodness-of-fit plots for the two covariate models (Left: AGE; Right: TRIAL)

| Full model:V_max_-WEIGHT K_a_-AGE V_1-_AGE V_max_-AGE | Full model:V_max_-WEIGHT K_a_-TRIAL V_1_- TRIAL V_max_- TRIAL |
| --- | --- |
| 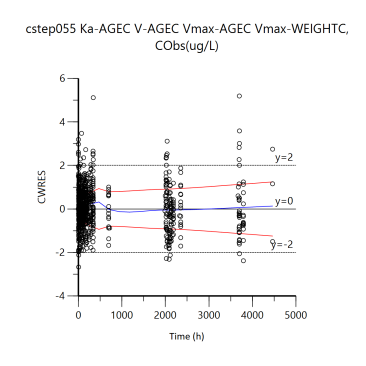 | 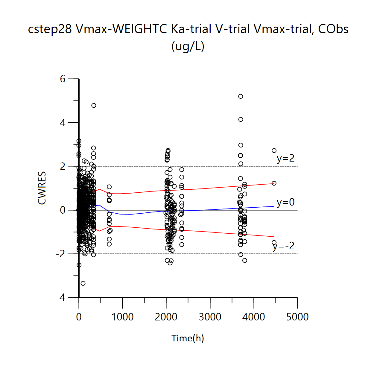 |
| 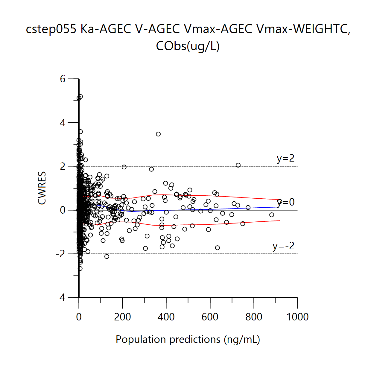 | 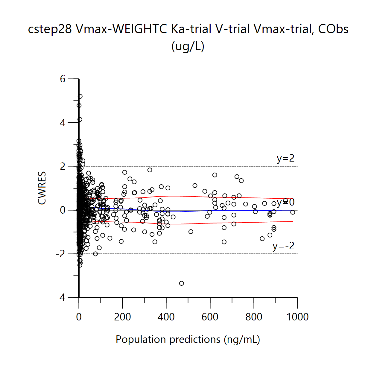 |
| 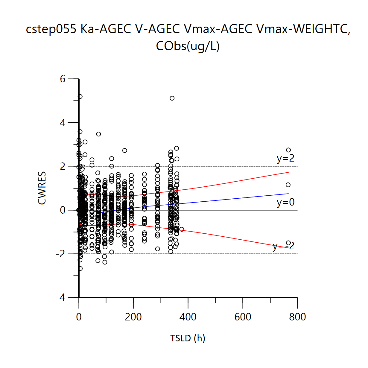 | 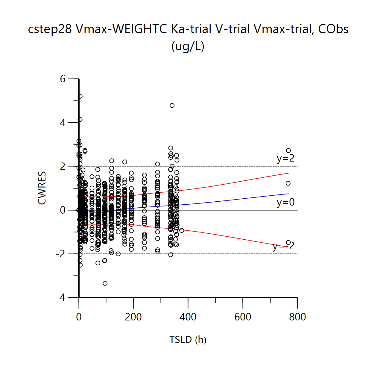 |
| 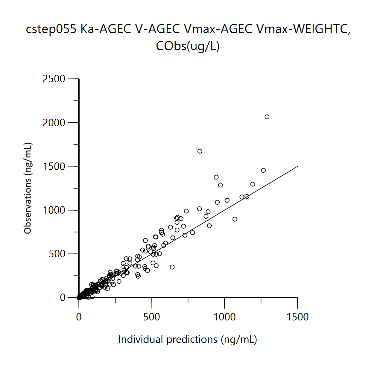 | 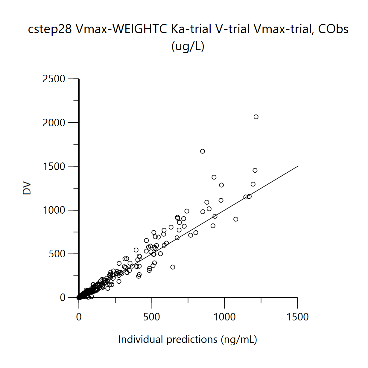 |
| 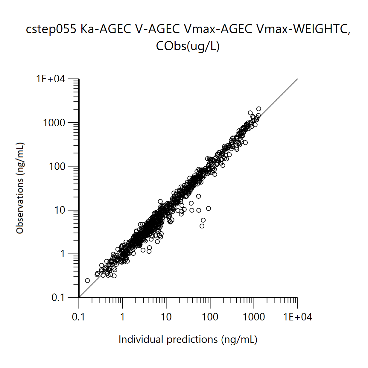 | 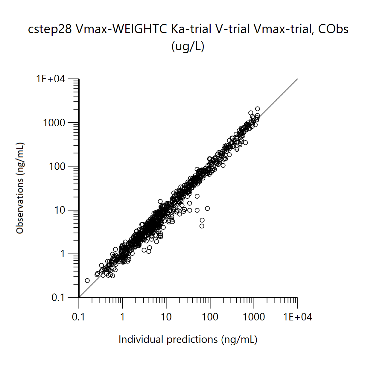 |
| 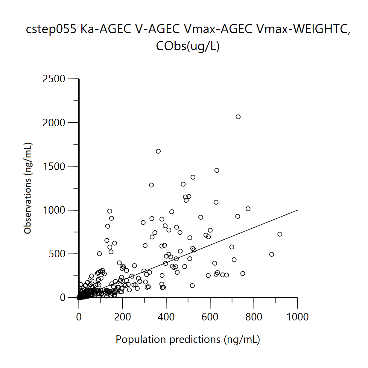 | 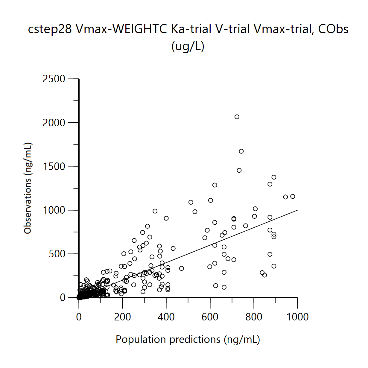 |
| 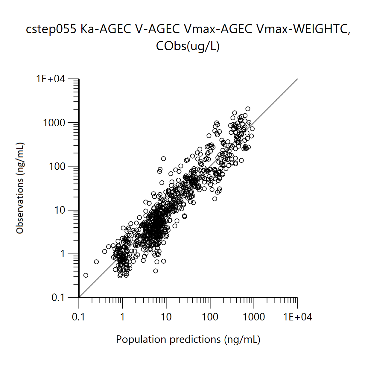 | 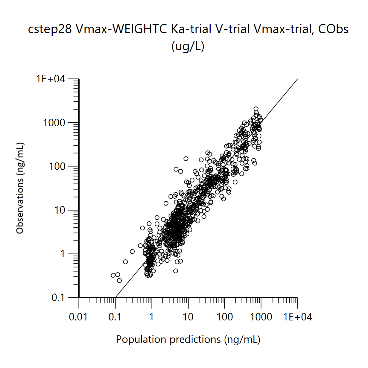 |

**Supplemental Figure 4:** Goodness-of-fit plots for the IDR model of IGF-1 relative-to-baseline ratios following YPEG-rhGH administration in healthy adult subjects. The top-left panel displays conditional weighted residuals (CWRES) versus time after the first dose. The top-middle and top-right panels show CWRES versus population predicted (PRED) and individual predicted (IPRED) values of IGF-1 relative-to-baseline ratios, respectively. The bottom-left and bottom-middle panels present observed IGF-1 relative-to-baseline ratios versus PRED and IPRED values, respectively. The bottom-right panel is a histogram of the probability density of CWRES. The red dashed lines represent the fitted trend lines.


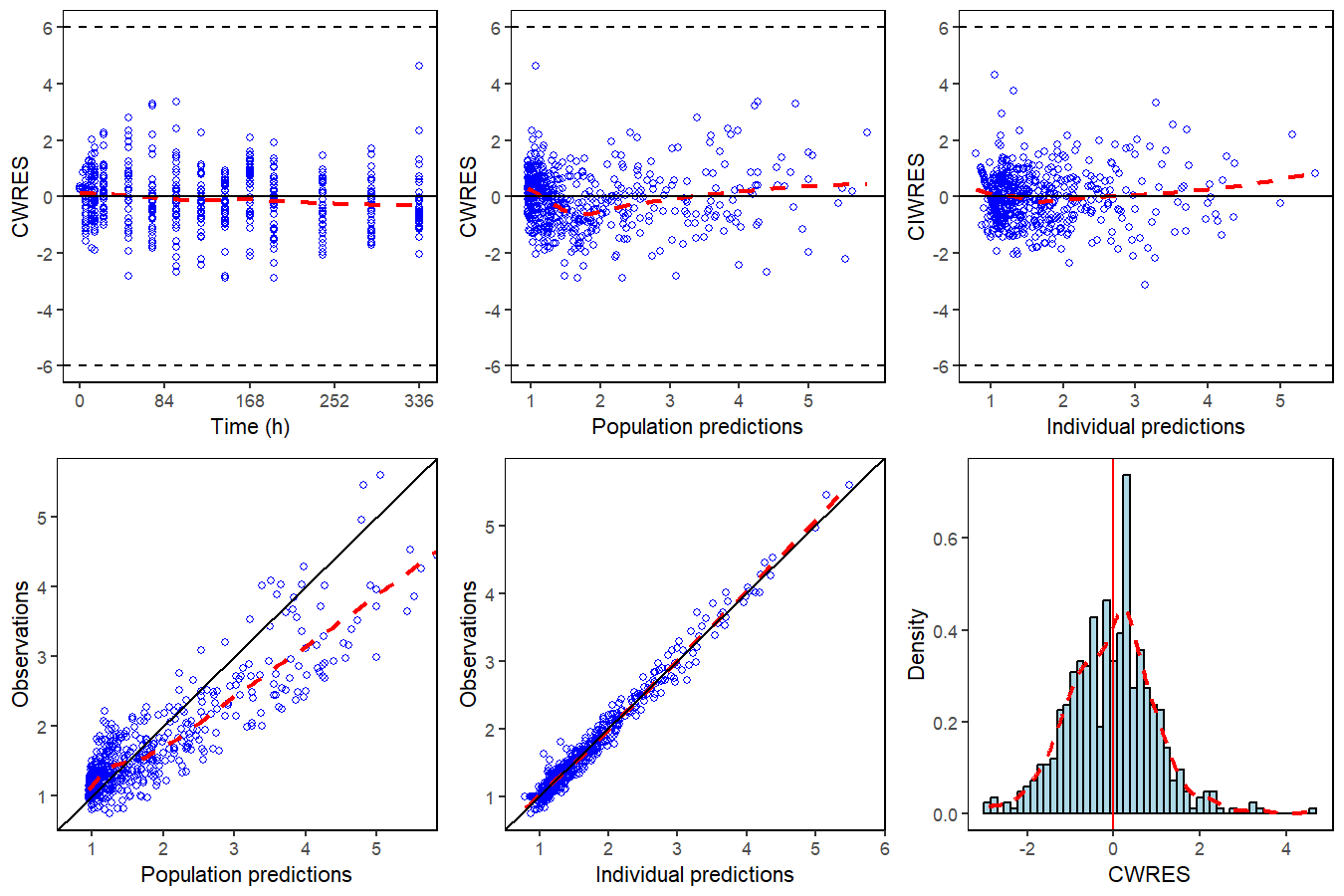


**Supplemental Figure 5:** Prediction-corrected visual predictive check (pvcVPC) for the IDR model of IGF-1 relative-to-baseline ratios following YPEG-rhGH administration in healthy adult subjects. Open circles represent the observed IGF-1 concentration ratios relative to baseline. The three lines from bottom to top correspond to the 10th, 50th (median), and 90th percentiles of the observed data, respectively. The shaded areas represent the 95% confidence intervals for the corresponding predicted percentiles.


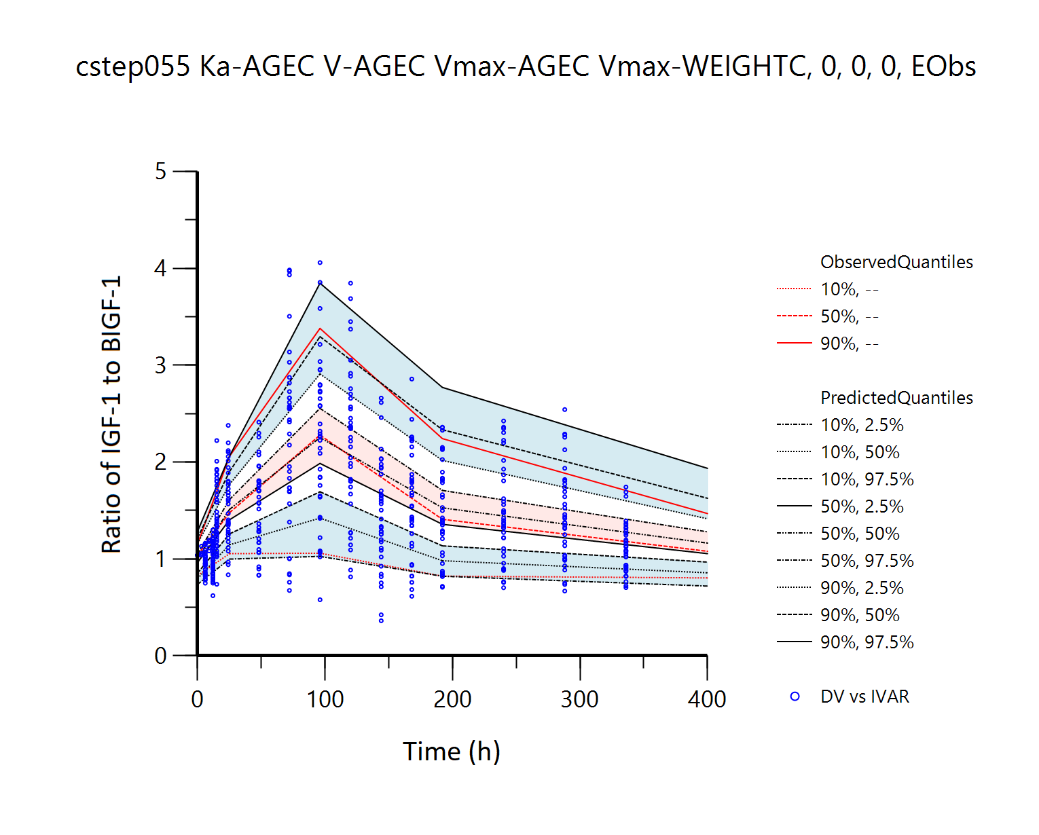


**Supplemental figure 6:** Diagnostic plots and individual fits of the PK/PD model using absolute IGF-1 values as the pharmacodynamic (PD) marker. The top panels show the model goodness-of-fit and predictive performance, including conditional weighted residuals (CWRES) versus time or predictions, observed versus individual predicted values, and population versus individual predictions. The bottom panels display individual fits with observed data points and model-predicted curves, demonstrating the model’s capability to describe the dynamic changes of IGF-1.

**
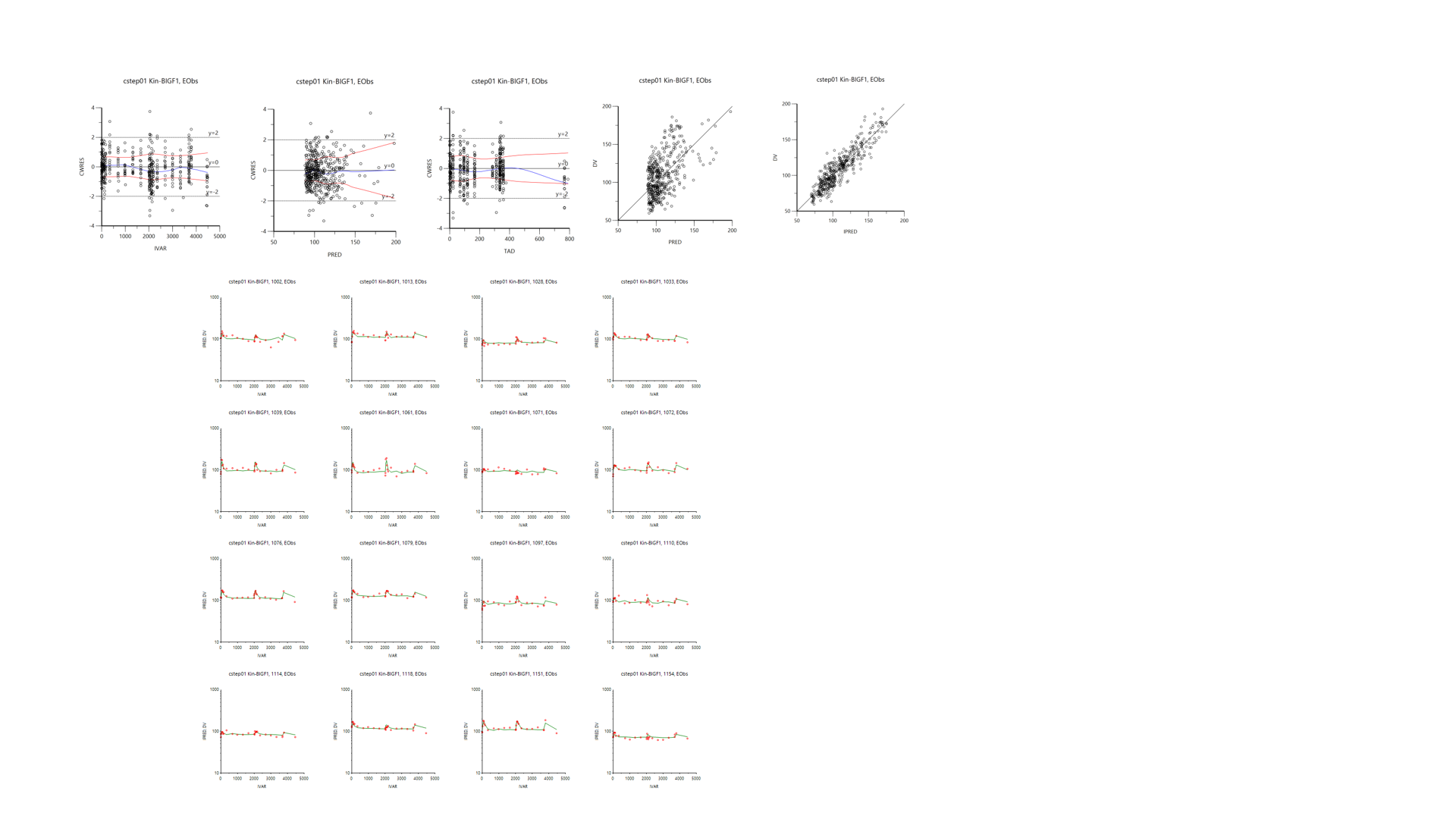
**

**PopPK model equations**

$$K_{a}(1/h)=0.01086\times\left( \frac{\mathrm{AGE}}{35.5} \right)^{-0.6957}$$

$$V_{1}/F(L)=2.387\times{(\frac{\mathrm{AGE}}{35.5})}^{1.470}$$

$$V_{2}/F\left( L \right)=21.42$$

$$K_{m}(\mu g/L)=70.08$$

$$V_{\max}\left( {\mu g}/h \right)=80.13\times\left( \frac{\mathrm{AGE}}{35.5} \right)^{0.7989}\times\left( \frac{\mathrm{WEIGHT}}{70} \right)^{2.202}$$

$$\mathrm{CL}_{2}/F\left( L/h \right)=0.05575$$

**PopPK/PD model equations**

$$\frac{dR_{\text{I}\text{GF-1}}}{\mathrm{dt}}=0.023\times(1+\frac{2.245\times C}{50.74+C})-0.023\times R_{\text{IGF-}1}$$
